# Non-canonical NF-κB drives a fate switch from germinal center to early effector B cells

**DOI:** 10.1016/j.isci.2026.116917

**Published:** 2026-07-22

**Authors:** Waqas Nawaz, Amos Fong, Miguel P. Cardoso, Jing Tang, Jenny Zhao, Michael Y. Li, Shinya Rai, Aidan Beresford, Waleed Alduaij, Merrill Boyle, Christian Steidl, David W. Scott, Leandro Venturutti

**Affiliations:** 1 Centre for Lymphoid Cancer, BC Cancer, Vancouver, BC, Canada; 2 Interdisciplinary Oncology Program, University of British Columbia, Vancouver, BC, Canada; 3 Department of Pathology and Laboratory Medicine, Faculty of Medicine, University of British Columbia, Vancouver, BC, Canada; 4 Department of Hematology and Rheumatology, Faculty of Medicine, Kindai University, Osaka-Sayama, Japan; 5 Department of Medicine, Faculty of Medicine, University of British Columbia, Vancouver, BC, Canada

**Keywords:** non-canonical NF-κB, B cells, adaptive immunity, vaccination, germinal center, plasma cells, regulatory T cells, IL-10

## Abstract

The non-canonical (NC) NF-κB pathway controls peripheral B-cell survival and follicular organization, pointing to a regulatory role during active adaptive immune responses. Here, we show that NC NF-κB activity is selectively attenuated in human germinal center B cells (GCBs), the population required to generate high-affinity antibodies, while remaining elevated in effector B cells. Accordingly, enforced NC NF-κB activation in murine B cells resulted in near-complete loss of GCB and high-affinity antibody production following immunization. These effects were primarily driven by NC NF-κB-induced reshaping of the immune microenvironment: activated B cells secreted IL-10, triggering premature expansion of regulatory T cells, limiting T cell help, and suppressing germinal center responses. Blocking IL-10 signaling was sufficient to normalize the niche and restore GCB. Concurrently, NC NF-κB activation promoted rapid B-cell differentiation into low-affinity antibody-secreting cells. Together, these findings identify NC NF-κB as a tunable rheostat linking B-cell signaling, immune-niche control, and humoral immunity.

## Highlights

• NC NF-κB signaling is selectively attenuated in germinal center B cells
• Sustained NC NF-κB activation suppresses germinal centers and affinity maturation
• B-cell-derived IL-10 expands regulatory T cells to reprogram the immune niche
• NC NF-κB signaling biases B cells toward a low-affinity plasma cell fate

## Graphical abstract

**Figure:**
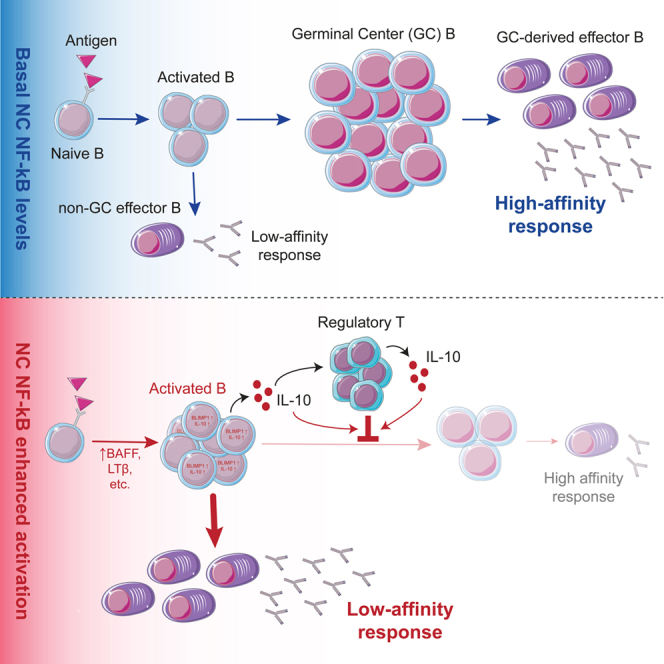

## Teaser

Molecular biology; Cell biology; Systems biology

## Introduction

The capacity to mount tailored and timely immune responses against diverse pathogens relies on the engagement of various specialized cell populations. B cells often play a pivotal role in these processes by mounting humoral responses, but also by shaping the behavior of other cells through cytokines and contact-mediated interactions.^1^ Most mature B cells reside in lymphoid follicles as follicular B cells (FOB), where they collaborate with CD4 T cells to respond to antigens with a protein component (“T-dependent (TD) responses”). Upon cognate antigen exposure, the majority of FOB undergo rapid proliferation bursts and differentiation into low-affinity antibody secreting cells (ASC), providing a first wave of protective immunity.^1^ A subset of activated FOB are instead diverted into germinal centers (GC), transient niches where they diversify their antigen receptor B cell receptor (BCR), and compete for T cell help, culminating in the production of high-affinity memory B cells (MBC) and plasma cells (PC).^2^ This intricate sequence of fate decisions requires tight temporal and spatial regulation, but also sufficient flexibility to adapt to evolving microenvironmental signals. Uncovering how such cues shape B-cell fate and function is critical to anticipating, and eventually manipulating, the magnitude and quality of these responses.

The NF-κB pathway acts as a central hub in B cells, integrating signals from diverse receptors to drive programs that support cell development, survival, activation, and effector functions.^3^ The pathway comprises two interconnected but non-redundant branches: canonical and non-canonical (NC), each triggered by distinct cues, involving specific mediators, and leading to the activation of different transcriptional regulators.^4^ The canonical branch is typically activated through the BCR, Toll-like receptors, or various cytokine receptors, and primarily involves the nuclear translocation of RelA/p50 dimers. Its contributions to B cell-driven responses have been extensively studied, including in cell survival, GC dynamics, and PC differentiation.^3^ Conversely, the NC arm is activated by a more restricted set of receptors (e.g., BAFF-R (B-cell activating factor receptor), LTβR), which cause the accumulation of the pathway’s central kinase NIK (NF-κB-inducing kinase) (*MAP3K14*), and nuclear shuttling of RelB/p52 dimers.^5^ While this branch is known to support B-cell maturation, survival, and follicular organization,^6^ a comprehensive description of its role in adaptive responses is missing. The biological relevance of the NC pathway can be inferred from the recurrent genetic alterations and dysregulated environmental cues (e.g., BAFF overexpression) causing its aberrant activation in mature B-cell malignancies (e.g., diffuse large B-cell lymphoma, multiple myeloma)^7^^,^^8^^,^^9^ and autoimmune disorders (e.g., systemic lupus erythematosus, Sjögren’s syndrome).^10^^,^^11^

Given NC NF-κB pathway’s growing significance in both homeostasis and disease, here we sought to investigate the dynamics and functional consequences of its activation in B cells during the time course of an adaptive immune response, aiming to resolve its cell-intrinsic roles and broader impact on immune system coordination.

## Results

### NC NF-κB is differentially activated across B-cell compartments

**Figure 1:**
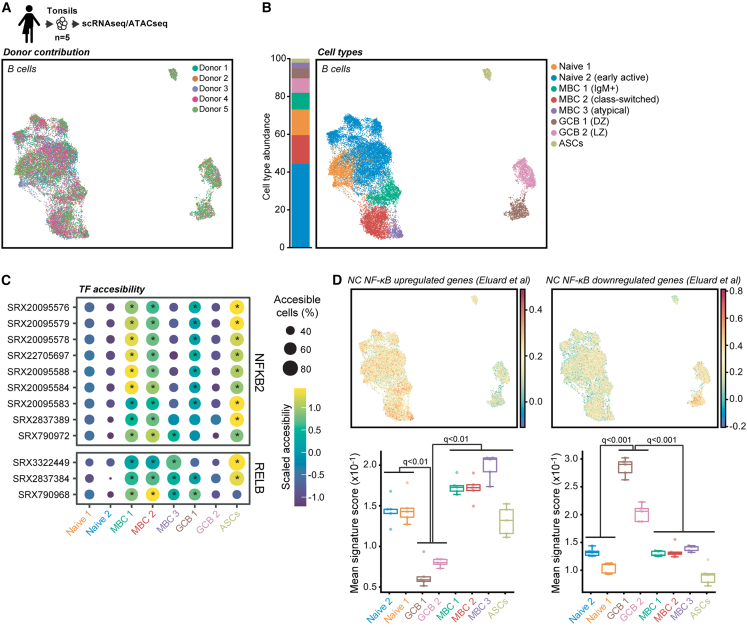
The NC NF-κB pathway is asymmetrically activated across human B cell populations (A) UMAP plot shows individual donor’s contribution to the single-nuclei multiome analysis of human tonsils. Non-B cells were excluded from the plot and analysis. (B) UMAP plot shows B-cell subsets detected in human tonsils from (A). The bar plot on the left represents relative abundances of each subgroup. See also Figure S1A. (C) Dot plot shows chromVAR assessment of accessibility in B cell subsets at functionally validated TF binding sites from the ChIP-Atlas.^12^ ∗q < 0.05, using one-tailed Welch *t* test with Benjamini-Hochberg correction. See also Table S1. (D) Top: UMAP plots show relative expression levels of genes (left) upregulated or (right) downregulated by the NC NF-κB pathway,^13^ in B cells in the multiome analysis. Bottom: Bar plots showing enrichment scores across B-cell subtypes from (B). Dots indicate individual tissue donors. Q values were calculated using a two-tailed Welch *t* test with Benjamini-Hochberg correction.

To identify stages of an adaptive immune response at which the modulation of NC NF-κB signaling may be functionally relevant, we first mapped the pathway’s status across human B-cell subsets. To this end, we performed single-nuclei multiome (snRNAseq/ATACseq) profiling of T cell (CD3)-depleted cell suspensions from human tonsils (*n* = 5) (Figure 1A), as prominent sites of B cell-driven adaptive responses.^14^ Integrated transcriptional and chromatin accessibility analysis of B cells (*n* = 20,857) identified eight distinct groups representing all major subsets involved in humoral responses. The four largest clusters corresponded to naive and MBC populations, with MBC further subdivided based on class-switch status as indicated by IgM (*IGHM*) expression (Figures 1B and S1A). Naive clusters showed asymmetric expression of broad activation markers (e.g., *CD44*, *CD69*), suggesting one of these groups captured an early activation state (Figure S1A). The remaining cells were sorted into two GC B cell (GCB) groups, an ASC group, and an atypical MBC group^15^ (Figures 1B and S1A). GCB subgroups captured established anatomical/functional regions in the GC, namely the dark zone (DZ) and light zone (LZ)^2^ (Figure S1A).

NC NF-κB activation typically culminates in nuclear shuttling of RelB and p52, which mediate the transcriptional effects of the pathway.^5^ To infer the binding activity of these transcription factors (TF) across B-cell subsets, we curated binding site feature sets from published ChIPseq datasets across multiple B-cell lines,^12^ and quantified chromatin accessibility at these sites using chromVAR^16^ (Table S1). Overall, effector B cells (MBC and ASC) exhibited the highest accessibility at RelB/p52 binding sites (Figure 1C), suggesting NC NF-κB is most active in these subsets. Naive B cells comparatively showed lower accessibility, with a relative increment in the early activated subset (Figure 1C). Unexpectedly, GCB exhibited markedly reduced accessibility at these sites (Figure 1C), particularly in the LZ compartment, indicating that NC NF-κB activity does not simply scale with activation or antigen exposure. The concordance between RelB and p52 accessibility patterns, and their reproducibility across feature sets derived from independent cell lines, underscored the robustness of this observation.

To validate these findings through an orthogonal approach, we queried the expression of NC NF-κB target genes, using functionally derived signatures from the literature. In line with the accessibility analysis (Figure 1C), effector B-cell populations showed strong enrichment scores for NC NF-κB upregulated genes,^13^ whereas both GCB clusters exhibited the lowest expression values (Figure 1D, left). Assessment of NC NF-κB downregulated genes^13^ revealed a concordant mirrored pattern (Figure 1D, right), with GCB clusters showing the highest levels of expression. In all, these data demonstrate that NC NF-κB is asymmetrically activated across B-cell populations, with selective attenuation in GCB, suggesting tight, compartment-specific control of pathway activity during adaptive immune responses.

### Augmented NC NF-κB activation is detrimental to GCB

The relatively low NC NF-κB activity observed among GCB (Figures 1C and 1D) pointed to a selective pressure differentially attenuating the cascade in this compartment. To functionally interrogate the impact of NC-sustained NF-κB activation, we leveraged a transgenic mouse model (*R26NIK*^*GFP*^)^17^ that enables Cre recombinase-inducible overexpression of the pathway’s central kinase, NIK. This genetic, stimulus-agnostic approach was chosen to isolate NC-specific effects, as ligand-driven stimulation (e.g., via LTβ, RANKL) can concomitantly engage the canonical NF-κB branch.^18^^,^^19^ In heterozygosis, the *R26NIK*^*GFP*^ transgene drives a moderate increase in signaling,^17^ maintaining levels within a physiologically relevant range.

**Figure 2:**
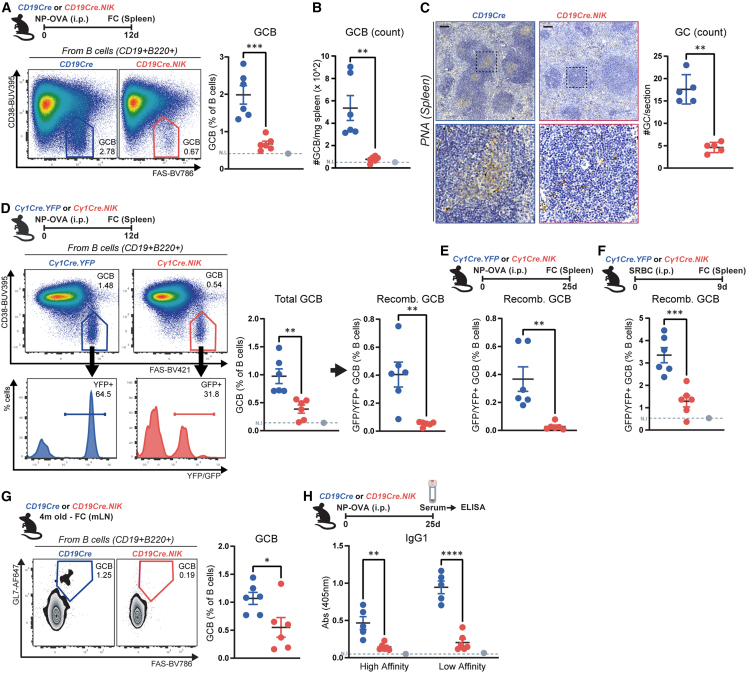
Augmented NC NF-κB activation is detrimental to GCB and GC-mediated humoral immunity (A and B) FC analysis of GCB, presented as (A) relative frequency or (B) cell numbers. N.I.: non-immunized control animal; *CD19Cre.NIK*: *CD19Cre;R26NIK*^*GFP*^. See also Figures S2A and S2B. (C) Peanut agglutinin (PNA) IHC in splenic sections from animals treated as in (A). Inserts show a zoom of outlined areas. Scale bars, 100 μm. Plot shows average GC numbers, where each dot is an individual animal. See also Figures S2C–S2E. (D) FC analysis of (left) total or (right) recombined GCB. *Cγ1Cre.YFP: Cγ1Cre;R26YFP; Cγ1Cre.NIK: Cγ1Cre;R26NIK*^*GFP*^. See also Figure S2F. (E and F) FC profiling of recombined GCB (E) at an alternative time point or (F) using an alternative antigen than in (D). (G) FC profiling of GCB in mesenteric lymph nodes (mLN) from treatment-naive animals. (H) ELISA results for NP-specific serum antibodies. Abs: absorbance. Individual dots represent biological replicates. Values represent mean ± SEM. Data were reproducible with two repeats. Here and elsewhere, value distributions were assessed using the Shapiro-Wilk normality test, and parametric or non-parametric statistical tests were applied accordingly. ∗*p* < 0.05; ∗∗*p* < 0.01; ∗∗∗*p* < 0.001, and ∗∗∗∗*p* < 0.0001, using unpaired two-tailed Student’s *t* test (A, C-F, H), or Mann-Whitney U-test (B, G).

To restrict enforced NC NF-κB activation to the B-cell compartment, we first crossed *R26NIK*^*GFP*^ mice to a *CD19Cre* strain.^20^ *CD19Cre;R26NIK*^*GFP*^ or age/sex-matched *CD19Cre* control mice were then immunized with the strictly TD antigen NP (4-Hydroxy-3-nitrophenylacetyl)-OVA, and profiled at the expected peak of the GC reaction (day 12).^21^ At this time point, *CD19Cre;R26NIK*^*GFP*^ mice showed a modest but significant expansion of total splenic B cells by flow cytometry (FC) (Figures S2A and S2B), consistent with previous reports in treatment-naïve animals.^17^ Histological analysis showed no evident disruption of splenic architecture or lymphoid follicles (Figure S2C). Strikingly, however, FC analysis revealed an almost complete lack of mature GCB (FAS+CD38-) in *CD19Cre;R26NIK*^*GFP*^ mice (Figures 2A and 2B). Comparable results were obtained using a broader phenotypic definition (FAS+GL7+), which further includes newly-formed and egressing GCB (Figure S2D), indicating a comprehensive loss of the GCB compartment. Concordantly, clusters of GCB (PNA+ cells) were readily detectable in follicles of immunized control mice, but were essentially absent in *CD19Cre;R26NIK*^*GFP*^ ones (Figure 2C). The GC deficit did not reflect a generalized failure of B-cell activation, as *CD19Cre;R26NIK*^*GFP*^ mice actually harbored an increased fraction of activated (IgD-) FOB compared to controls (Figure S2E).

Since the early constitutive activation of the NC pathway in *CD19Cre*-based models could introduce potential confounder effects, we next restricted NIK overexpression to activated FOB. To this end, we crossed *R26NIK*^*GFP*^ mice to a *Cgamma1Cre* strain^22^ (*Cγ1Cre),* resulting in NC NF-κB activation from a GCB precursor stage. Immunization of *Cγ1Cre;R26NIK*^*GFP*^ mice with NP-OVA similarly resulted in a marked reduction in total GCB abundance compared to *Cγ1Cre* controls (Figure 2D). To determine if the residual GCB represented recombination escapees, we assessed GFP expression from the *R26NIK*^*GFP*^ cassette and compared it to YFP (yellow fluorescent protein) expression from a CRE-dependent *R26YFP* reporter^23^ bred into *Cγ1Cre* control mice. When analysis was restricted to recombined (GFP+ or YFP+) GCB, the NC NF-κB-driven loss was nearly complete (Figures 2D and S2F). Hence, increased NC NF-κB signaling exerted similar effects on GCB, regardless of whether it was induced before or after FOB activation. *Cγ1Cre;R26NIK*^*GFP*^ mice continued to display a profound deficit in recombined GCB 25 days after immunization (Figure 2E), a time when GC reactions are resolving in control animals,^21^ arguing against delayed kinetics as an explanation for the observed phenotype.

To determine if findings could be generalized to other TD antigens, we conducted immunizations with sheep red blood cells (SRBC), which similarly resulted in a pronounced GCB deficit in *Cγ1Cre;R26NIK*^*GFP*^ mice (Figure 2F). In addition, examination of mesenteric lymph nodes sites of chronic GC activity driven by dietary antigens and commensal microbes^24^ revealed a major reduction in GCB in treatment-naive *CD19Cre;R26NIK*^*GFP*^ mice (Figure 2G). Collectively, these results demonstrate that augmented NC NF-κB activation is broadly deleterious to GCB across antigens, tissues, and modes of induction.

### Augmented NC NF-κB activation compromises GC-mediated humoral immunity

A central outcome of the GC reaction is the production of high-affinity antibodies. Given the profound GCB loss induced by enhanced NC NF-κB signaling, we next evaluated the impact on humoral immune quality. Circulating anti-NP antibodies were measured by ELISA (enzyme-linked immunosorbent assay) in *Cγ1Cre;R26NIK*^*GFP*^ and control mice 25 days after NP-OVA immunization. As expected,^25^ control mice mounted robust low- and high-affinity IgG1 responses at this time point (Figure 2H). In contrast, *Cγ1Cre;R26NIK*^*GFP*^ mice exhibited severely diminished IgG1 anti-NP titers, closer to non-immunized animals (Figure 2H). These findings indicate that sustained NC NF-κB activation in B cells profoundly compromises GC-dependent affinity maturation and humoral immune quality.

### NC NF-κB enhanced activation does not intrinsically impede GCB differentiation

**Figure 3:**
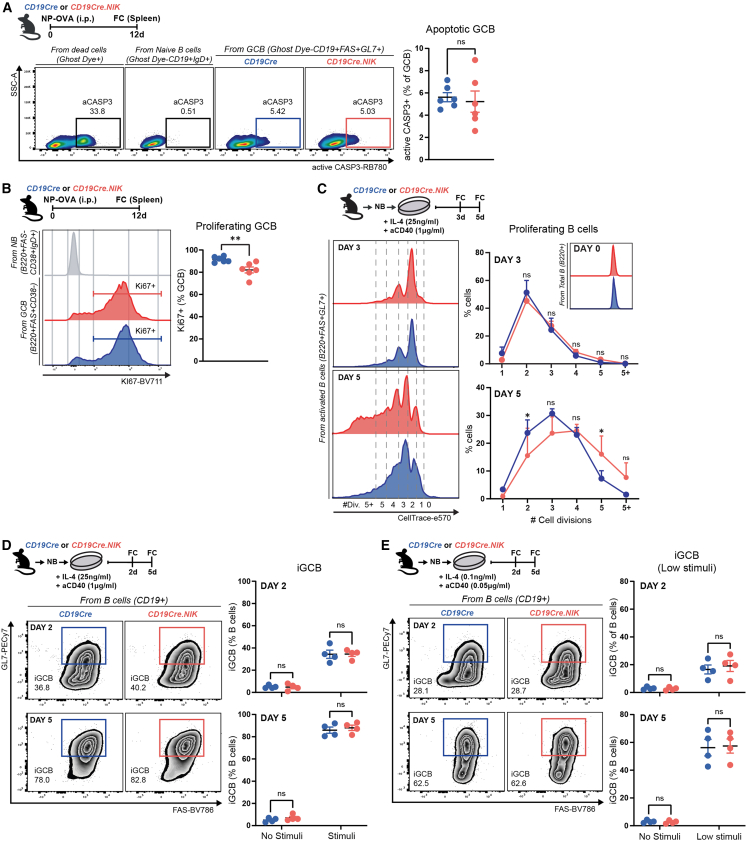
NC NF-κB-induced GCB loss is not driven by cell intrinsic effects (A) FC analysis of apoptotic GCB abundance, based on intracellular active caspase-3 expression. Results in dead cells and live naive B (NB) cells are shown as positive and negative staining controls, respectively. See also Figure S3A. (B) FC analysis of proliferating GCB abundance, based on intracellular Ki67 expression. NB illustrates non-proliferating cells. See also Figure S3B. (C) FC analysis of activated B cells *ex vivo* proliferation, based on dilution of a cell trace dye. Representative plots for day 0 are included as an insert to illustrate initial dye loading. (D) FC analysis of iGCB formation with standard stimulation. See also Figures S3C and S3D. (E) FC analysis of iGCB formation with sub-optimal stimulation. Individual dots represent biological replicates. Values represent mean ± SEM. Data are reproducible with two repeats. NS, not significant; ∗*p* < 0.05; ∗∗*p* < 0.01; ∗∗∗*p* < 0.001, using unpaired two-tailed Student’s *t* test (A, B), two-way ANOVA (C), or Mann-Whitney U-test (D and E).

We next sought to define the mechanisms underlying the NC NF-κB-induced GCB loss. During an active immune response, GC size is largely governed by a balance between proliferation and apoptosis.^2^ Because most residual GCB in *CD19Cre;R26NIK*^*GFP*^ mice were recombined, as indicated by GFP expression (Figure S3A), we focused our analyses on this population. At the peak of the GC reaction, the frequency of apoptotic GCB, assessed by active caspase-3 staining, was comparable between NIK-overexpressing and control cells (Figure 3A), indicating that excessive cell death did not account for the near-complete loss of GCB.

On the other hand, NC NF-κB induction was associated with a modest reduction in the fraction of proliferating GCB, as measured by Ki67 or PCNA staining (Figures 3B and S3B). However, the limited magnitude of this effect appeared insufficient, on its own, to explain the severity of the GCB phenotype in *CD19Cre;R26NIK*^*GFP*^ mice. To further assess whether NC NF-κB activation intrinsically impairs B cell proliferative capacity, we conducted *ex vivo* assays where naive FOB were loaded with a trace dye, activated, and profiled by FC over time. The extent of dye dilution, a proxy for cell division, was comparable between activated B cells from *CD19Cre;R26NIK*^*GFP*^ or control mice early on (Figure 3C, top), with NIK-overexpressing cells drawing a mild cumulative advantage over time (Figure 3C, bottom). Thus, enhanced NC NF-κB activation does not intrinsically limit B cell proliferation.

Given the negligible differences in proliferation and cell death, we hypothesized that increased NC NF-κB activation might instead antagonize the differentiation of FOB into GCB. In spite of the contraction of the total GCB pool, the frequency of precursor GCB^26^ among activated B cells was comparable in NIK-overexpressing and control mice (Figure S3C), suggesting any potential blockage should occur past this early fate commitment. To directly assess differentiation capacity, we adapted an *ex vivo* culture system^27^ in which naive FOB receive unrestricted co-stimulation via CD40/IL-4, prompting them to acquire a GCB-like phenotype (“iGCB.”) The frequency of iGCB generated over time was comparable between genotypes (Figure 3D), and iGCB from both genotypes upregulated the master TF BCL6^28^ to a similar extent (Figure S3D). Because saturating co-stimulation could mask more subtle defects, we repeated these experiments using lower stimulus concentrations, which resulted in sub-optimal iGCB formation from control cells (Figure 3E). Even under these settings, enforced NC NF-κB activation did not impair iGCB formation relative to controls (Figure 3E). Together, these data indicate that sustained NC NF-κB signaling does not intrinsically impair activation, proliferation, or differentiation toward a GCB fate, suggesting that the GC defects arise through cell-extrinsic mechanisms.

### NC NF-κB augmented activation in B cells causes a regulatory T cell expansion

**Figure 4:**
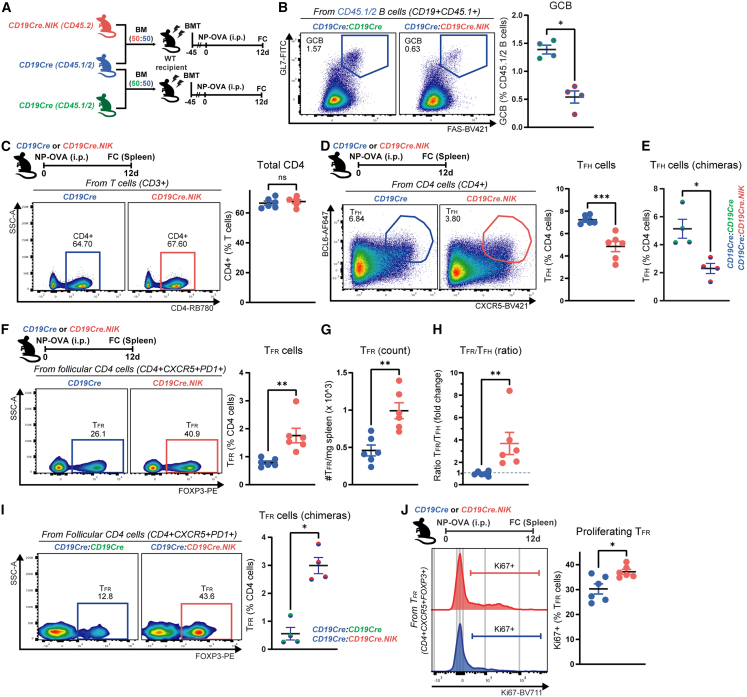
Augmented NC NF-κB activation in B cells drives expansion of regulatory T cells (A) Experimental scheme for (B), (E), and (I). (B) FC analysis of GCB derived from CD45.1+ B cells in chimeric mice. (C and D) FC profiling of (B) total CD4 or (C) T_FH_ cells abundance. See also Figures S4A–S4D. (E) FC analysis of T_FH_ cells in chimeric mice. (F–H) FC analysis of T_FR_ cells, presented as (F) relative frequency, (G) cell numbers, or (H) the normalized ratio between T_FR_ and T_FH_ cells in each animal. See also Figures S4E–S4G. (I) FC analysis of T_FR_ cells in chimeric mice. (J) FC analysis of proliferating T_FR_ abundance, based on intracellular Ki67 expression. Individual dots represent biological replicates. Values represent mean ± SEM. Data are reproducible with two repeats. NS, not significant; ∗*p* < 0.05; ∗∗*p* < 0.01; ∗∗∗*p* < 0.001, using Mann-Whitney U-test (B, E, H, I), or unpaired two-tailed Student’s *t* test (C, D, F, G, J).

The absence of intrinsic defects prompted us to investigate whether NC NF-κB activation in B cells suppresses GC responses by altering the immune niche. To this end, we generated mixed bone marrow chimeras reconstituted with equal proportions of control B cells (*CD19Cre;CD45.1/2*) and either NIK-overexpressing (*CD19Cre;R26NIK*^*GFP*^*;CD45.2*) or additional control (*CD19Cre;CD45.2*) B cells (Figure 4A). In this setting, B cells from both donors shape and share the same immune niche. Following reconstitution and immunization, chimeric mice were profiled at the expected peak of the GC response (Figure 4A). Notably, *CD19Cre;CD45.1/2* control B cells generated significantly fewer GCB when coexisting with NIK-overexpressing cells than when paired with other control cells (Figure 4B). These findings demonstrate that NC NF-κB-activated B cells dominantly suppress GC formation in trans, implicating soluble or regulatory mediators within the shared niche.

T follicular helper cells (T_FH_) offer critical support to GCB, and the duration and quality of their interactions largely dictate the fate of the latter.^29^ *CD19Cre;R26NIK*^*GFP*^ mice presented normal levels of CD4 T cells (Figure 4C), but a significant reduction in T_FH_ at the peak of the GC reaction (Figure 4D). A similar reduction was observed in immunized mixed chimeras carrying NIK-overexpressing B cells (Figure 4E), and in mice where NIK overexpression was restricted to activated FOB (Figures S4A and S4B). The expansion and activity of T_FH_ is heavily influenced by B cells and by the suppressive effects of follicular regulatory T cells (T_FR_).^30^ Residual GCB in immunized *CD19Cre;R26NIK*^*GFP*^ mice expressed MHCII and the co-stimulatory protein CD86 (Figures S4C and S4D) in similar proportions to WT GCB, suggesting preserved capacity to engage T cells. In contrast, NC NF-κB activation induced a pronounced accumulation of T_FR_ (Figures 4F–4H), which were scarce in control mice, as expected. T_FR_ expansion was also evident in the *Cγ1Cre*-dependent model (Figure S4E), and following immunization with alternative TD antigens (Figure S4F), supporting the generality of these findings. This was also the case for immunized mixed chimeras carrying NIK-overexpressing B cells (Figure 4I), highlighting the dominant effect of NC NF-κB activation on the immune niche. Importantly, T_FR_ accumulation was detectable from the earliest stages of the response (Figure S4G), before GC formation,^21^ and was associated with increased proliferation (Ki67+) within the compartment (Figure 4J). Together, these findings indicate NC NF-κB activation in B cells reprograms the immune niche by promoting an early and sustained expansion of suppressive T_FR_, which could account for the observed inhibitory effects on GC-mediated immune responses.

### IL-10 mediates the GC loss induced by NC NF-κB-enhanced activation in B cells

**Figure 5:**
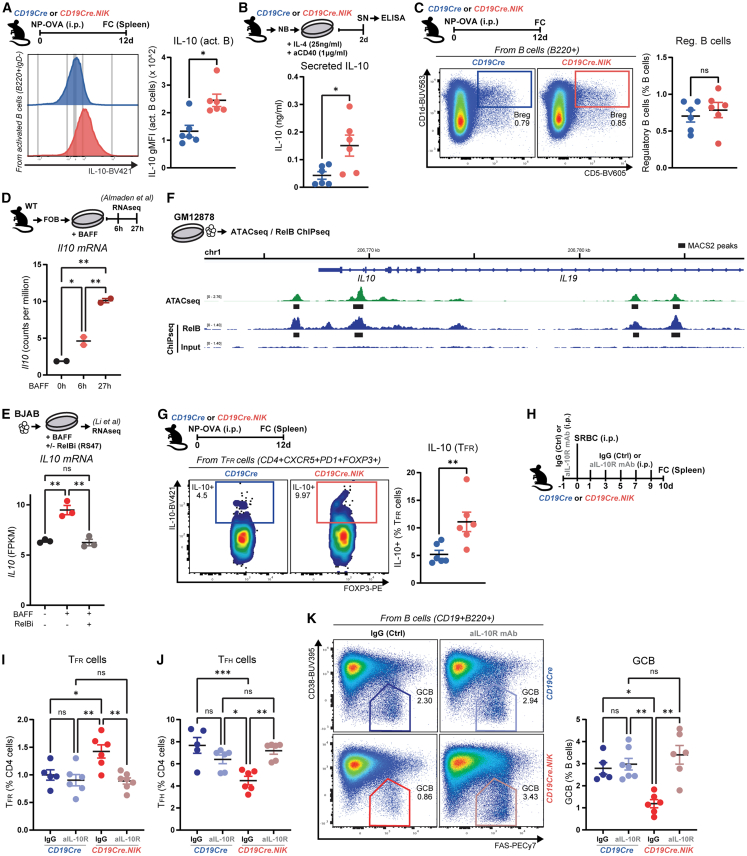
IL-10 mediates GC loss driven by NC NF-κB hyperactivation in B cells (A) FC analysis of intracellular IL-10 expression in activated B cells. Geometric mean fluorescence intensity (gMFI). (B) ELISA results for IL-10 in the conditioned supernatant from *ex vivo* cultured FOB. (C) FC analysis of regulatory B cells relative abundance. (D) RNAseq analysis of *Il10* mRNA expression levels in the indicated model.^33^ (E) RNAseq analysis of *IL10* mRNA expression levels in the indicated model (Li et al., 2025). Fragments per kilobase million (FPKM). (F) ChIPseq analysis of RelB binding at the *IL10* locus.^34^ An ATACseq track is included to indicate accessible regions in the same model. Representative tracks are shown. Black boxes indicate significant peaks called by MACS2.^35^ (G) FC analysis of intracellular IL-10 expression in T_FR_ cells. (H) Experimental scheme for I-K. (I and K) FC analysis of (I) T_FR_ cells, (J) T_FH_ cells, or (K) GCB, in animals treated as indicated in (H). Individual dots represent biological replicates. Values represent mean ± SEM. Data are reproducible with two repeats. NS, not significant; ∗*p* < 0.05; ∗∗*p* < 0.01; ∗∗∗*p* < 0.001, using Mann-Whitney U-test (A), unpaired two-tailed Student’s *t* test (B, C, G), one-way ANOVA with Tukey’s post-test (D, E), or two-way ANOVA with Tukey’s post-test (I-K).

IL-10 is a key cytokine involved in the formation and function of regulatory T cells.^31^^,^^32^ Based on our previous observations that oncogenic NC NF-κB activation in malignant B cells induced IL-10 expression,^7^ we investigated whether a similar mechanism operates during physiological immune responses. As expected, in immunized control mice, IL-10 expression was nearly undetectable in activated B cells (Figure 5A). Conversely, activated B cells in *CD19Cre;R26NIK*^*GFP*^ mice exhibited robust upregulation of intracellular IL-10 (Figure 5A), and IL-10 accumulated in the conditioned supernatant of NIK-overexpressing FOB cultured *ex vivo* (Figure 5B). The abundance of classic regulatory B cells (CD1d+CD5+)^36^ -known producers of IL-10^37^- was unchanged (Figure 5C), suggesting IL-10 induction represents a direct consequence of NC NF-κB signaling, rather than a readout for the expansion of this subpopulation.

To validate these findings using orthogonal approaches, we queried publicly available transcriptomic datasets. BAFF stimulation of WT murine FOB^33^ induced a progressive increase in *Il10* mRNA expression (Figure 5D). Similarly, BAFF-treated human BJAB cells^38^ upregulated *IL10* transcripts (Figure 5E), an effect reversed by pharmacologic inhibition of RelB. Consistent with direct transcriptional control, ENCODE ChIPseq data from human lymphoblastoid B cells with constitutive NC NF-κB activation (GM12878)^34^ revealed basal RelB binding at accessible regions within the *IL10* locus (Figure 5F). Together, these data support a model in which NC NF-κB signaling directly induces IL-10 expression in B cells.

In addition to B cells, IL-10 is commonly produced by regulatory T cell subsets. IL-10R signaling has been shown to further facilitate IL-10 production by these T_FR_.^32^ In line with the expanded T_FR_ pool in NC NF-κB-activated mice (Figures 4F and 4G), a higher fraction of T_FR_ expressed detectable IL-10 protein compared to controls (Figure 5G). These findings suggest that NC NF-κB-driven IL-10 production by B cells initiates a feedforward loop, in which expanded T_FR_ further amplify IL-10 levels within the GC niche.

To directly test whether IL-10 signaling is required for the observed niche-mediated suppression of GC responses, we performed IL-10 receptor blockade experiments. Mice were immunized with SRBC and treated with an IL-10R-blocking or isotype-control antibody, beginning one day prior to immunization and continuing every other day (Figure 5H). IL-10R blockage was sufficient to prevent the NC NF-κB-driven expansion of T_FR_ cells (Figure 5I). This treatment also rescued the NC NF-κB-induced contraction of the T_FH_ pool (Figure 5J). More importantly, IL-10R blockage was enough to restore GCB abundance in *CD19Cre;R26NIK*^*GFP*^ mice (Figure 5K). These results demonstrate that IL-10 signaling is required for NC NF-κB-dependent immune-niche reprogramming and suppression of GC-mediated immunity.

### NC NF-κB activation promotes the accumulation of low-affinity ASC

**Figure 6:**
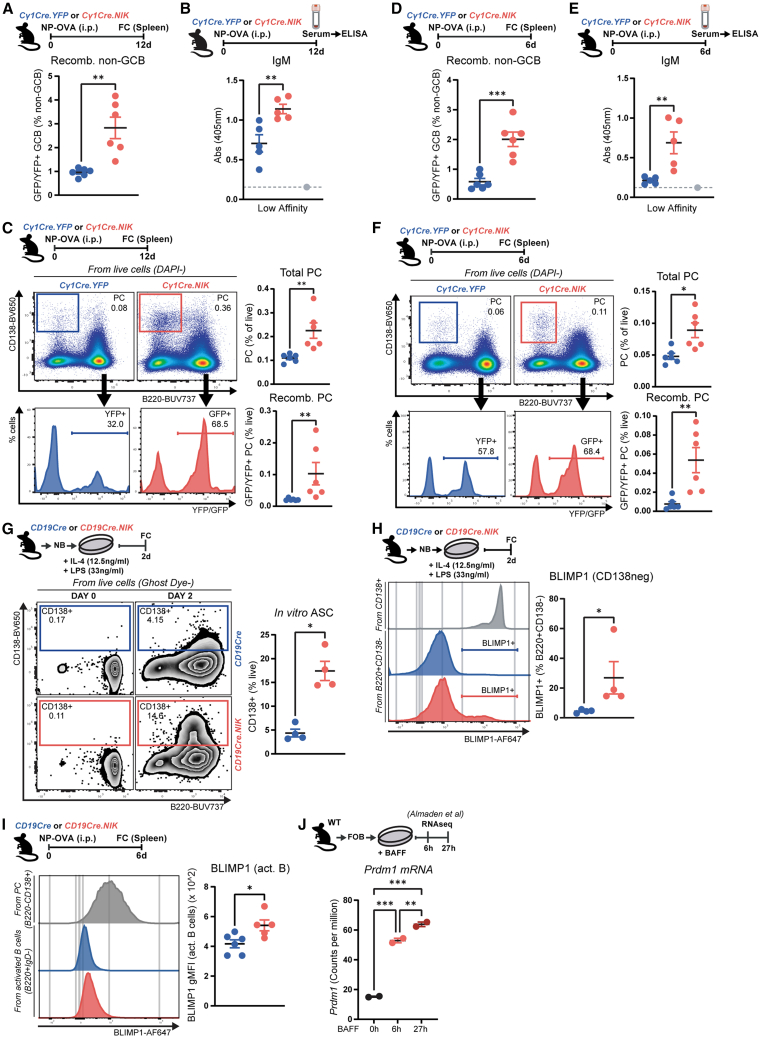
NC NF-κB activation facilitates the accumulation of low-affinity ASCs (A) FC analysis of recombined (GFP/YFP+) non-GCB cells at the expected peak of the reaction. See also Figure S5A. (B) ELISA results for NP-specific serum antibodies from animals treated as in (A). (C) FC analysis of total and recombined (GFP/YFP+) PC from animals treated as in (A). See also Figures S5B and S6C. (D) FC analysis of recombined (GFP/YFP+) non-GCB cells at an early time point in the reaction. (E) ELISA results for NP-specific serum antibodies from animals treated as in (D). See also Figures S5E and S5F. (F) FC analysis of total and recombined (GFP/YFP+) PC from animals treated as in (D). (G) FC profiling of *in vitro*-derived ASC. See also Figure S5G. (H) FC analysis of intracellular BLIMP1 expression in CD138-negative cells from the experiment in (G). Staining results in CD138+ cells are shown as a positive control. See also Figures S5H–S5I. (I) FC analysis of intracellular BLIMP1 expression in activated B cells. Staining results in PC are shown as a positive control. (J) RNAseq analysis of *Prdm1* expression levels in the indicated model.^33^ See also Figures S5J–S5L. Individual dots represent biological replicates. Values represent mean ± SEM. Data are reproducible with two repeats. ∗*p* < 0.05, ∗∗*p* < 0.01, and ∗∗∗*p* < 0.001, using unpaired two-tailed Student’s *t* test (A, D), Mann-Whitney U-test (B, C, E–I), one-way ANOVA with Tukey’s post-test (J).

In TD responses, the default fate for activated FOB is the formation of GC-independent low-affinity ASC, whereas only a minority enter the GC pathway.^1^ Given the elevated NC NF-κB activity observed in human effector B-cell populations relative to GCB (Figures 1C and 1D), we asked whether NC NF-κB activation not only suppressed GC responses but also actively promoted an effector cell fate. Consistent with this hypothesis, immunized *Cγ1Cre;R26NIK*^*GFP*^ mice -despite failing to generate GC responses (Figure 2D)- displayed a significant expansion of recombined non-GCB cells (Figure 6A). ELISA on serum from these animals revealed an accumulation of low-affinity anti-NP IgM antibodies (Figure 6B), indicative of enhanced ASC output. Indeed, FC analysis confirmed a robust accumulation of recombined PC in *Cγ1Cre;R26NIK*^*GFP*^ mice, 12 days after immunization (Figure 6C). In contrast, the non-recombined PC pool size remained comparable across genotypes (Figure S5A), demonstrating that this effect was specifically driven by NC NF-κB activation. A similar expansion of total PC was observed in immunized *CD19Cre;R26NIK*^*GFP*^ mice (Figures S5B and S5C).

Notably, differences in recombined PC abundance and antibody titers in *Cγ1Cre;R26NIK*^*GFP*^ mice were detectable from an early time point (Figures 6D–6F), when the GC-independent route is the largest contributor to the humoral response.^21^ Significant differences persisted for at least 25 days post-immunization (Figure S5D), a stage when most high-affinity GC-derived PC have been produced in control mice.^21^ Together, these findings indicate that NC NF-κB activation compromises affinity maturation while driving a quantitatively enhanced, low-affinity humoral response.

Low-affinity humoral responses are most commonly elicited by T-independent antigens. These are also GC-independent responses, typically driven by marginal zone B cells outside the follicle.^1^ To explore if NC NF-κB activation could further boost these responses, we immunized *CD19Cre;R26NIK*^*GFP*^ or control mice with NP-LPS, and profiled the response at its expected peak.^39^ Indeed, augmented NC NF-κB activation resulted in a disproportionate accumulation of NP-specific splenic plasmablasts (Figure S5E), and circulating low-affinity anti-NP antibodies (Figure S5F), concordant with an enhanced response. In all, these findings show that the activation of the NC NF-κB pathway broadly amplifies GC-independent humoral responses.

### NC NF-κB activation facilitates the formation of ASC

The early and robust expansion of PC suggested NC NF-κB signaling lowers the threshold for plasmacytic differentiation. To directly test this, we isolated NIK-overexpressing or control FOB from treatment-naive mice and cultured them *ex vivo* in the presence of LPS to promote ASC differentiation. In line with our *in vivo* findings, FC analysis revealed a disproportionate accumulation of CD138+ cells derived from NIK-overexpressing B cells (Figure 6G), consistent with an intrinsic propensity toward ASC differentiation. Notably, even under iGCB culture conditions, we observed an incipient but reproducible increase in CD138+ cells in the NIK-overexpressing group (Figure S5G), suggesting that NC NF-κB activation broadly biases B-cell fate decisions toward effector differentiation.

Since stimulatory conditions in the assays above were identical for both genotypes, the ASC expansion was likely supported by intrinsic changes introduced by NC NF-κB activation. The TF BLIMP1 (*PRDM1*) is the master regulator of plasmacytic differentiation,^40^ and its expression is induced as B cells commit to this fate. As expected, *in vitro*-derived CD138+ cells from both genotypes expressed elevated BLIMP1 levels (Figure S5H). Notably, a differential fraction of NIK-overexpressing CD138- B cells also had detectable levels of BLIMP1 (Figure 6H), indicating premature commitment to a PC fate. Consistent with these findings, *in vivo* analysis revealed disproportionate BLIMP1 upregulation among activated B cells in immunized *CD19Cre;R26NIK*^*GFP*^ mice early in the response (Figure 6I).

Re-analysis of published RNAseq data^33^ further showed acute BAFF treatment induces rapid and progressive *Prdm1* upregulation in WT FOB (Figure 6J). Furthermore, ChIPseq data from GM12878 cells revealed basal RelB occupancy at the *PRDM1* locus (Figure S5I), supporting direct transcriptional regulation downstream of NC NF-κB signaling. Collectively, these findings indicate that NC NF-κB activation intrinsically primes B cells toward plasmacytic differentiation, diverting them away from GC programs.

### NC NF-κB activation does not promote atypical B cell fates

The canonical NF-κB cascade facilitates B-cell differentiation into diverse fates,^41^ including the formation of so-called aged/autoimmune/atypical B cells (AiBC)^42^ through both GC-dependent^43^ and GC-independent^44^^,^^45^ routes. Given the potent impact of NC NF-κB on B-cell differentiation demonstrated here, we investigated whether this axis also facilitated the expansion of AiBC. Notably, *CD19Cre;R26NIK*^*GFP*^ mice showed a moderate but significant contraction of the AiBC pool (defined as T-BET+CD11c+ MBC; Figure S5J). Similar results were obtained using CD11b as an alternative marker to define this population (Figure S5K). To validate these findings through an orthogonal approach, we queried mRNA expression of the same core AiBC markers in WT FOB stimulated *ex vivo* with BAFF.^33^ As expected, basal expression of these genes was relatively low. Consistent with our *in vivo* results, we observed further downregulation of *Itgax* (CD11c) and *Tbx21* (T-BET) following BAFF treatment, while *Itgam* (CD11b) levels remained largely unchanged (Figure S5K). Together, these data suggest that NC NF-κB activation is not a primary driver of AiBC formation.

## Discussion

In this study, we sought to refine current models of B-cell-mediated immunity by mapping the activation of the NC NF-κB pathway across human B-cell subsets, and coupling these findings with targeted functional studies. Our findings reveal that NC NF-κB signaling exerts a dynamic, context-dependent regulatory influence on adaptive humoral responses, extending well beyond its established role in B-cell survival.

Previous studies demonstrated that GCB fundamentally depend on NF-κB signaling for their survival, as genetic ablation of pathway components (e.g., NIK, RelB, p52)^46^^,^^47^ compromises GC responses. Similarly, antigen-experienced B cells from patients with a NIK deficiency display reduced BCR diversity and class-switching,^48^ two GC-associated features. Together, these observations support a long-standing model in which basal NC NF-κB signaling is permissive and required for GC biology. Our findings introduce a more complex and seemingly paradoxical scenario: While some NC NF-κB activity is necessary, excessive activation is detrimental to GC responses. Human GCB displayed detectable yet attenuated NC NF-κB activity relative to other activated B-cell subsets, suggesting that pathway output is finely tuned to meet regulatory requirements, rather than simply switched on or off. This attenuation may represent a protective constraint that shields the GC reaction from the pathological consequences of hyperactivated NC signaling. A similar mechanism regulates the canonical NF-κB stream, where downregulation of the inhibitory protein A20 is needed for B-cell activation, but its complete loss leads to deleterious outcomes,^49^ thus setting maximum tolerable activation levels for that pathway.

Changes in the activation levels of other signaling pathways in GCB (e.g., BCR, canonical NF-κB, mTOR) have been shown to exert their core effects through cell-intrinsic mechanisms.^50^^,^^51^^,^^52^ Similarly, NC NF-κB blockage was found to compromise GC responses by inducing transcriptional changes in B cells that hinder co-stimulation and cell cycle progression.^46^^,^^47^ In alignment with this model, a previous study had proposed that NC NF-κB activation could impair the GC reaction through a B-cell-intrinsic defect in BCL6 upregulation.^8^ However, BCL6 was only modestly downregulated *in vivo*, and its overexpression failed to alter the GC phenotype.^8^ Our findings further argue against this model by showing that NIK-overexpressing B cells could still adopt a GCB phenotype *ex vivo* and express normal BCL6 levels. Instead, we found that enforced NF-κB activation dominantly reprograms the immune niche, suppressing GC formation in trans. Other cell-intrinsic effects, including the strong PC cell fate bias, could further contribute to the observed phenotypes by concurrently redirecting activated B cells away from the GC reaction. However, the relative influence of such mechanisms is likely asymmetric, as modulation of the milieu was sufficient to normalize the niche and virtually rescue the GCB pool.

One of the most striking findings of our study is the identification of IL-10 as the key mediator of NC NF-κB-driven niche regulation and GC suppression. IL-10 plays a dual role in GC biology: it supports GC maintenance under physiological conditions,^53^ while acting as a negative feedback regulator to prevent exacerbated responses.^54^ Although T_FR_ are the principal source of IL-10 in established GC reactions, additional contributions arise from T_FH_, regulatory B cells, and stromal cells.^55^^,^^56^^,^^57^ Our findings reveal that aberrantly elevated NC NF-κB activity triggers IL-10 production by activated B cells, initiating a feedforward regulatory circuit that expands T_FR_ cells, suppresses T_FH_ support, and prevents GC establishment. Indeed, IL-10R blockade normalized the immune niche and restored GC B-cell abundance, demonstrating that IL-10 signaling is required for NC NF-κB-driven GC suppression. These observations underscore how the timing and cellular source of IL-10 critically shape its functional outcome. Notably, canonical NF-κB signaling can also induce IL-10 in B cells,^58^ yet B cell-specific hyperactivation of this pathway does not suppress GC responses.^43^ This difference may be explained by the fact that, unlike NC signaling, canonical NF-κB activation concurrently hypersensitizes B cells to co-stimulatory cues, and lowers the threshold for GCB formation and expansion.^43^ It should be noted that, while our data points to IL-10 as the primary modulator of the observed phenotypes, the interplay between B and T cells is multifaceted, and likely involves additional secondary signals working in concert downstream of NC NF-κB activation.

Our findings also provide insight into disease contexts characterized by aberrant NC NF-κB activation. We previously showed that oncogenic NC NF-κB signaling in B-cell lymphomas induces IL-10 production and suppresses anti-tumor immunity.^7^ Our data reveal that, rather than a *de novo* feature of malignancy, NC NF-κB already regulates this circuitry in normal cells, and that this is instead hijacked and re-purposed by malignant B cells. This constitutes yet another example of transformed B cells exploiting physiological regulatory mechanisms.^59^

Beyond GC suppression, our work identified NC NF-κB as a critical determinant of activated B-cell fate. Prior studies in aged and repeatedly immunized NC NF-κB-activated mice had also documented a PC expansion.^8^ Our work shows a single immunization was sufficient to drive an early and robust PC accumulation, pointing to a GC-independent differentiation trajectory. In support of this model, NC NF-κB activation also exacerbated the GC-independent response to a T-independent antigen. Furthermore, we show that enhanced NC NF-κB activation lowered the threshold for PC fate adoption through a cell-intrinsic mechanism. From a physiological perspective, this signaling logic may be adaptive. Several viruses and bacteria are known to activate the NC NF-κB pathway in the host's immune cells.^60^^,^^61^ A transient NC NF-κB-driven switch from high-affinity, lower-output to a low-affinity, high-output antibody response could provide timely control of initial pathogen load, sacrificing specificity for speed. This trade-off, while beneficial during acute infections, means pathogens may not be fully neutralized, and could further carry risks if chronically engaged or unbalanced.

Our findings also address the relationship between NC NF-κB signaling and the generation of AiBC. The NC NF-κB pathway is found aberrantly activated in autoimmune disorders such as systemic lupus erythematosus, where AiBC play a central pathogenic role.^62^ Interestingly, our data show NC NF-κB activation does not drive the *de novo* induction of AiBC. Instead, NC NF-κB may modulate the pathology of these diseases by exacerbating the differentiation of existing AiBC into antibody-secreting cells and/or by remodeling the immune niche composition to favor inflammation.

In all, our findings invite a revision of current models, placing NC NF-κB not merely as a permissive signal, but as a pivotal driver -and at times, limiter- of B cell function and fate. These insights open avenues for selectively modulating B-cell responses in vaccination, autoimmunity, and cancer.

### Limitations of the study

Our IL-10R blockade experiments provide strong functional evidence for the importance of this pathway, but do not allow us to dissect the specific contributions of individual IL-10R-expressing cell types. Similarly, our current data establish that B-cell-derived IL-10 is necessary to initiate the observed effects, but do not test whether this is sufficient to drive the observed phenotypes, or whether this relies instead on a feedforward loop driven by regulatory T cells and/or other IL-10-producing populations in the niche. Our study primarily examined NC NF-κB function in vaccination settings using model antigens. Future work is required to determine how NC NF-κB signaling shapes B-cell responses during live infections or in the context of complex, persistent antigens. In addition, the long-term implications of NC NF-κB activation on immune memory and protective recall responses remain untested. Finally, while our genetic models enabled the isolation of NC NF-κB-specific effects, ligands can often engage multiple pathways simultaneously. Dissecting how these integrated inputs collectively shape immune outcomes will be an important area for future investigation.

## Resource availability

### Lead contact

Requests for further information and resources should be directed to and will be fulfilled by the lead contact, Leandro Ventrutti (lventurutti@bccrc.ca).

### Materials availability

All materials and reagents, and data generated in this article are available upon request to the lead contact. Detailed protocols for experiments reported in this article are available upon request to the lead contact.

### Data and code availability

• Multiome sequencing data have been deposited at The European Genome-phenome Archive under study accession number EGAS50000001646 and are available as of the date of publication. Because these data contain sensitive human information, access is controlled to protect participant privacy. To request access, researchers must apply through the EGA portal (https://ega-archive.org/studies/EGAS50000001646); access requires the completion of a standardized Data Access Agreement between the requesting and hosting institutions.
• Gene expression data (read counts) from BAFF-treated murine FOB^33^ were retrieved from the Gene Expression Omnibus (GSE62559).
• Gene expression data (read counts) from BAFF-treated murine BJAB cells^38^ were retrieved from the Gene Expression Omnibus (GSE288086).
• ChIPseq data (MACS2 peak calls and tracks) from treatment-naive GM12878 cells was downloaded from the ChIP-Atlas^12^ (SRX3322448; SRX2423908; SRX10478546).
• This paper does not report original code. Code used for analysis is available upon request.
• Any additional information required to reanalyze the data reported in this paper is available from the lead contact upon request.

## Acknowledgments

We thank all members of the Venturutti Lab for thoughtful discussions and suggestions, and Hisae Nakamura for outstanding project management. We acknowledge BC Cancer’s Animal Resource Center (ARC), Flow Cytometry Core Facility (FCCF), and the Histology and Digital Imaging Core (BASIC). Funding: BC Cancer Foundation Rising Stars Fellowship (W.N., A.F., J.Z.), Canada Graduate Research Scholarship (J.Z.), 10.13039/100009447Leukemia & Lymphoma Society of Canada and Blood Cancer United Translational Research Program #6663-23 (C.S./L.V.), Michael Smith Health Research Foundation Scholarship SCH-2022-2607 (L.V.), 10.13039/501100015803BC Cancer Foundation Startup Funds (L.V.), Canada Foundation for Innovation John R. Evans Leaders Fund #43630 and Innovation Fund #46036 (L.V.), 10.13039/501100000024Canadian Institutes of Health Research Project #180613 (L.V.), 10.13039/501100004376Terry Fox Research Institute Program Project #1108 (C.S.). C.S. has performed consultancy for Bayer.

## Author contributions

Conceptualization: L.V., C.S, W.N.; methodology: W.N., A.F., D.W.S., L.V.; software: A.F.; investigation: W.N., A.F., M.P.C., J.T., J.Z., S.R., A.B., W.A., M.B.; resources: L.V., M.Y.L., D.W.S., C.S.; writing: L.V., W.N.; visualization: W.N., A.F., L.V.; funding acquisition: L.V., C.S.

## Declaration of interests

The authors declare no competing interests.

## STAR★Methods

### Key resources table

**Table:** 

REAGENT or RESOURCE	SOURCE	IDENTIFIER
**Antibodies**		

BB700 anti-mouse CD19	BD Biosciences	Cat#566411; RRID: AB_2744315
BUV737 anti-mouse CD45R/B220	BD Biosciences	Cat#612838; RRID: AB_2870160
PE-Cy7 anti-mouse CD95	BD Biosciences	Cat#557653; RRID: AB_396768
FITC anti-mouse T- and B-Cell Activation Antigen	BD Biosciences	Cat# 553666; RRID: AB_394981
BV786 anti-mouse IgD	BD Biosciences	Cat#*563618,* RRID: AB_2738322
R718 anti-mouse CD45	BD Biosciences	Cat#567075; RRID: AB_2916421
BV711 anti-mouse Ki-67	BD Biosciences	Cat#*563755;* RRID: AB_2738406.
BV786 anti-mouse CD95	BD Biosciences	Cat#*740906;* RRID: AB_3684328
BUV395 anti-mouse CD38	BD Biosciences	Cat#*740245;* RRID: AB_2739992.
RB780 anti-Active Caspase-3	BD Biosciences	Cat#*570335;* RRID: AB_3685672
RB780 anti-mouse CD4	BD Biosciences	Cat#*568694,* RRID: AB_3676036
BUV563 anti-mouse CD3	BD Biosciences	Cat#*749277;* RRID: AB_368532
BV421 anti-mouse CD185	BD Biosciences	Cat#*562889;* RRID:AB_536012
Rat anti-mouse CD16/CD32 (Fc block)	BD Biosciences	Cat# 553142; RRID:AB_394657
BV421 anti-mouse CD185	BD Biosciences	Cat#*562889;* RRID:AB_536012
Biotin anti-mouse CD185	BD Biosciences	Cat# *551960;* RRID: AB_394301
PE-Cy7 anti-mouse CD25	BD Biosciences	Cat#552880; RRID: AB_394509
BUV805 anti-mouse I-A/I-E	BD Biosciences	Cat# 748844; RRID: AB_2873247
BV421 anti-mouse IL-10	BD Biosciences	Cat# *563276;* RRID: AB_2738111
BUV563 anti-mouse CD1d	BD Biosciences	Cat#741287; RRID: AB_2870821
BV605 anti-mouse CD5	BD Biosciences	Cat#*563194;* RRID: AB_2738061
BV650 anti-mouse CD138	BD Biosciences	Cat#*564068;* RRID: AB_2738574
BUV737 anti-mouse Blimp-1	BD Biosciences	Cat#567834; RRID: AB_3683930
BUV563 anti-mouse CD23	BD Biosciences	Cat#*741228;* RRID: AB_2870782
BUV737 anti-mouse CD21/CD35	BD Biosciences	Cat#612810; RRID: AB_2870135
BUV661 Rat Anti-CD11b	BD Biosciences	Cat# 612977; RRID:AB_2870249
PE-Cy7 anti-mouse/human GL7 Antigen	BioLegend	Cat# 144620; RRID: AB_2563314
Alexa Fluor 647 anti-mouse/human Bcl-6 Antibody	BioLegend	Cat# 648305; RRID: AB_2562039
BV605 anti-mouse CD86	BioLegend	Cat# 105037; RRID: AB_11204086
AF647 Streptavidin	BioLegend	Cat# 405237
PE anti-human/mouse/rat PCNA	BioLegend	Cat# 307908; RRID: AB_493313
APC/Cyanine7 anti-mouse CD11c Antibody	Biolegend	Cat# 117324; RRID:AB_830649
PE/Cyanine5 anti-T-bet Antibody	Biolegend	Cat# 644841; RRID:AB_3662326
APC anti-mouse CD4	Thermo Fisher Scientific	Cat# 17-0041-81; RRID: AB_469320
PerCP-Cy5.5 anti-mouse FOXP3	Thermo Fisher Scientific	Cat# 45-5773-82; RRID: AB_914351
eFluor 450 anti-mouse IRF4	Thermo Fisher Scientific	Cat# 48-9858-82; RRID: AB_10853346
FITC anti-mouse CD279 (PD-1)	Thermo Fisher Scientific	Cat# 11-9985-85; RRID: AB_465514
PE-Cy7 Streptavidin	Thermo Fisher Scientific	Cat# 25-4317-82; RRID:AB_10116480
Ephrin-B1 anti-mouse Biotinylated Antibody	R&D Systems, a Bio-Techne brand	Cat# BAF473; RRID:AB_2293418
NP-PE (4-Hydroxy-3-nitrophenylacetyl hapten conjugated to R-PE)	Biosearch Technologies	Cat# N-5070-1
Ghost Dye™ Violet 510 Viability Dye	Tonbo Biosciences	Cat# 13-0870-T500
DAPI (4′,6-Diamidino-2-Phenylindole, Dihydrochloride)	Thermo Fisher Scientific	Cat# D3571; RRID: AB_2307445
Polyclonal Armenian hamster IgG	BioXCell	Cat# BE0091; RRID:AB_1107773
InVivoMab anti-mouse IL-10R (CD210) antibody (clone 1B1.3A)	Bio X Cell	Cat# BE0050; RRID: AB_1107594
Peanut Agglutinin (PNA), Biotinylated	Vector Laboratories	Cat# B-1075; RRID: AB_2313597
CD19 Monoclonal Antibody (6OMP31), eBioscience™	Thermo Fisher Scientific	Cat# 14-0194; RRID: AB_467209
PerCP-Cy5.5 anti-human CD3 Antibody (clone OKT3)	Thermo Fisher Scientific (eBioscience™)	Cat# 45-0036-42; RRID: AB_11070005
BD Horizon™ BV480 Mouse Anti-Human CD3	BD Biosciences	Cat# 566105; RRID: AB_2739519

**Biological samples**		

Sheep red blood cells	Rockland Immunochemicals; Limerick, PA, USA	Cat# R311-0050

**Chemicals, peptides, and recombinant proteins**		

NP-OVAL	Biosearch Technologies	Cat# N-5051
NP-KLH	Biosearch Technologies	Cat# N-5060
NP-LPS	Biosearch Technologies	Cat# N-5065
alhydrogel® adjuvant 2%	InvivoGen, San Diego, CA, USA	Cat#vac-alu-50
Recombinant Murine IL-4 Protein	R&D Systems, a Bio-Techne brand	Cat# 404-ML; RRID: AB_345489
Functional Grade Purified anti-mouse CD40 Antibody (clone 1C10)	Thermo Fisher Scientific (Invitrogen™ eBioscience™)	Cat# 16-0402-82; RRID: AB_468927
Lipopolysaccharides from *E. coli* O111:B4	MilliporeSigma (Merck)	Cat# L2630

**Critical commercial assays**		

Foxp3/Transcription Factor Staining Buffer Set	ThermoFisher Scientific	Cat#501128857
CountBright Plus Absolute Counting Beads	ThermoFisher Scientific	Cat#C36995
EasySep Mouse B Cell Isolation Kit	StemCell Technologies	Cat#19854C
eBioscience™ Cell Proliferation Dye eFluor™ 670	Thermo Fisher Scientific	Cat# 65-0840-85
DAB Peroxidase (HRP) Substrate Kit	Vector Laboratories	Cat# SK-4100
SBA Clonotyping System	Southern Biotechnology	Cat# 5300-05
ELISA MAX™ Standard Set Mouse IL-10	BioLegend	Cat# 431411
Chromium Next GEM Chip J Single Cell Kit	10× Genomics	Cat# 1000234
Chromium Next GEM Single Cell Multiome ATAC + Gene Expression Reagent Bundle	10× Genomics	Cat# 1000283
Chromium i7 Multiplex Kit N, Set A	10× Genomics	Cat# 1000212
Chromium i7 Multiplex Kit TT, Set A	10× Genomics	Cat# 1000215

**Deposited data**		

Multiome sequencing data from human tonsils	This paper	European Genome Phenome Archive: EGAS50000001646
Transcriptional profiling of BAFF-treated murine FOB	Almaden et al.^33^	Gene Expression Omnibus: GSE62559
Transcriptional profiling of BAFF-treated BJAB cells	Li et al.^38^	Gene Expression Omnibus: GSE288086
ChIPseq and ATACseq data from treatment-naive GM12878 cells	ChIP-Atlas/ENCODE	SRX3322448; SRX2423908; SRX10478546

**Experimental models: Organisms/strains**		

Mouse: C57BL/6J	The Jackson Laboratory	Stock# 000664; RRID: IMSR_JAX:000664
C57BL/6-Gt(ROSA)26Sortm5(Map3k14)Rsky/J (*R26NIK*^*GFP*^)		Stock# 012637; RRID: IMSR_JAX:012637
Mouse: *Cγ1-Cre* (B6.129P2(Cg)-Ighg1tm1^(cre)Cgn^/J)	The Jackson Laboratory	Stock# 010611; RRID: IMSR_JAX:010611
Mouse *CD19-Cre* (B6.129P2(C)-Cd19^tm1(cre)Cgn^/J)	The Jackson Laboratory	Stock# 006785; RRID: IMSR_JAX:006785
Mouse: CD45.1 (B6.SJL-Ptprc^a^Pepc^b^/Boy)	The Jackson Laboratory	Stock# 002014; RRID: IMSR_JAX:002014
Mouse: Rosa26-lox-stop-lox-YFP (B6.129X1-Gt(ROSA)26Sor^tm1(EYFP)Cos^/J)	The Jackson Laboratory	Stock# 006148; RRID: IMSR_JAX:006148

**Software and algorithms**		

FlowJo	Becton Dickinson	Version 10.5.3
GraphPad Prism	GraphPad Software	Version 10.1.2
Aperio eSlide Manager	Leica Biosystems	https://www.leicabiosystems.com/aperio-eslide-manager/
Fiji (ImageJ)	Kalampokis et al.^56^	https://imagej.net/Fiji
Cell Ranger ARC (v2.0.2)	10× Genomics	Software suite
Python (v3.11)	Python Software Foundation	

**Other**		

Graphical Abstract Cartoons	SERVIER Medical Art Repository	https://smart.servier.com

### Experimental model and study participant details

#### Mouse models

Animal breeding and experimental procedures were approved by The University of British Columbia’s Animal Care Committee (protocols A22-0104 and A21-0205), and conducted in strict compliance with The Canadian Council on Animal Care guidelines. The following strains were obtained from The Jackson Laboratory (Ben Harbor, ME, USA): C57Bl/6J (strain 000664), C57BL/6-Gt(ROSA)26Sortm5(Map3k14)Rsky/J (*R26NIK*^*GFP*^, strain 012637), Cγ-1Cre (strain 010611), CD19-Cre (strain 006785), B6.SJL-PtprcaPepcb/Boy (CD45.1, strain 002014) and Rosa26-lox-stop-lox-YFP (*R26YFP*; strain 006148). Animals were housed in a specific-pathogen free facility at the BC Cancer Research Institute. Experiments were conducted using aged/sex-matched littermates whenever possible. Experiments included male and female animals, and no sex bias was detected in the observations made in this work. Unless stated otherwise, animals were 8-12 weeks of age at the time of experimentation. Bone marrow transplants to generate chimeric animals were conducted as previously described.^63^ Briefly, bone marrow cells were collected from the tibia and femur of donor mice, treated with RBC lysis solution (158904; QIAGEN; Germantown, MD, USA), mixed at the indicated ratios, and injected i.v. into lethally irradiated C57BL/6J recipient mice. Transplanted mice were used for experiments 6-8 weeks after transplant, to allow for full engraftment.

#### Human participants

Sourcing and profiling of human tonsillar specimens was conducted in accordance with the Declaration of Helsinki, and approved by the University of British Columbia - BC Cancer Research Ethics Board (Protocol H18-00469). Cell isolation was performed on five archived human reactive tonsil samples from the BC Cancer Biobank. Donor’s sex and age at collection: Donor 1 = Male, 5 years; Donor 2 = Male, 15 years; Donor 3 = Male, 29 years; Donor 4 = Female, 23 years; Donor 5 = Female, 21 years.

### Method details

#### Immunizations and *in vivo* treatments

Mice were immunized i.p. with 250 μL of a 2% SRBC suspension (R311-0050; Rockland Immunochemicals; Limerick, PA, USA) prepared in saline, or 100 μg of a highly substituted NP conjugate (NP_16_ to NP_32_) to ovalbumin (OVA) or Keyhole Limpet Hemocyanin (KLH) (Biosearch Technologies, Novato, CA, USA), absorbed to alhydrogel adjuvant 2% (vac-alu-50; InvivoGen, San Diego, CA, USA) at a 1:1 ratio. In experiments studying T cell-independent responses, animals were immunized i.p. with 25 μg of NP-LPS prepared in saline (Biosearch Technologies). In experiments where IL-10 signaling was blocked, mice received i.p. injections of 200 μg anti-IL10R antibody (clone 1B1.3A, BE0050; BioXCell, West Lebanon, NH, USA) or IgG control antibody (BE0091; BioXCell). In these experiments, animals were randomly assigned to treatment (random number generator).

#### Histology and immunohistochemistry

Organs were fixed in 4% formaldehyde. Embedding, sectioning and staining were conducted by the BASIC Lab at BC Cancer (Vancouver, BC, Canada), as described.^43^ The following primary antibodies were used for IHC: anti-PNA (B1075; Vector Laboratories, Newark, CA, USA) and anti-CD19 (14-0194; ThermoFisher Scientific, Waltham, MA, USA). Images were captured using a Zeiss Axioscan 7 scanner (Zeiss, Oberkochen, Germany). Scanned images were examined using Aperio eSlide Manager (Leica Biosystems; Wetzlar, Germany), and Fiji software^64^ was used to count PNA-positive clusters within follicles to quantify GC. Images were converted to 8-bit grayscale, thresholded to highlight PNA-positive regions, and analyzed using the ‘Analyze Particles’ function with size thresholds set to exclude background staining. GC counts were reported per section, and all image processing and counting parameters were applied identically across all samples.

#### Primary B-cell cultures

Splenocyte suspensions from treatment-naive mice were pre-enriched via negative selection, using an EasySep Mouse B-Cell Isolation Kit (19854; StemCell Technologies, Vancouver, BC, Canada). Resulting cell suspensions were subjected to cell sorting, for the isolation of naive FOB (DAPI-CD19+CD23^hi^CD21^int^FAS-GL7-IgD + CD138-). Where indicated, cells were stained with eBioscience Cell Proliferation Dye eFluor 670 (65-0840-85; ThermoFisher Scientific), following the vendor’s protocol. Cells were then seeded at 1 × 10ˆ6 cells/mL in RPMI media with 10% FBS (35-077-CV; Corning, Corning, NY, USA), penicillin G/streptomycin, MEM non-essential AA, 2-Mercaptoethanol (all from ThermoFisher Scientific). For iGCB induction, complete media was supplemented with murine recombinant IL-4 (404-ML; R&D Systems, Minneapolis, MN, USA) and a functional-grade agonistic anti-CD40 antibody (16–0402-82; ThermoFisher Scientific), added at graded concentrations: standard (25 ng/mL IL-4 and 1 μg/mL anti-CD40) or low (0.1 ng/mL IL-4 and 0.05 μg/mL anti-CD40). For iPC induction, complete media was supplemented with 33 ng/mL LPS (L2630; MilliporeSigma, Burlington, MA, USA) and 12.5 ng/mL murine recombinant IL-4. Cells were incubated at 37°C with 5% CO2 and culture medium was renewed every 2–3 days.

#### ELISA

Conditioned supernatants were collected at the indicated timepoints after last culture media exchange, spun down to separate from the cell pellet, and filtered to remove any residual cells. Serum from animals was collected at the stated days following immunization. Low and high-affinity NP-specific titers were measured in plates coated with NP_32_-BSA or NP_9_-BSA (Biosearch Technologies), respectively, using an SBA Clonotyping System (5300-05; Southern Biotechnology; Birmingham, AL, USA). Murine IL-10 titers were measured using an ELISA MAX Standard Set (431411; BioLegend, San Diego, CA, USA), following the vendor’s protocol. Results were assessed by spectrophotometric measurement of absorbance at 405 nm (NP-BSA assays) or 450 nm (IL-10 assays) using a Spark Multimode Microplate Reader (Tecan, Männedorf, Switzerland).

#### Flow cytometry and cell sorting

Cell suspensions were pre-treated with mouse Fc Block (553142; BD Biosciences, San Josa, CA, USA) and stained with the indicated antibodies. From BD Biosciences: BB700 anti-mouse CD19 (566411; dilution 1:500), BUV737 anti-mouse CD45R/B220 (612838; dilution 1:500), PE-Cy7 anti-mouse CD95 (557653; dilution 1:500), FITC anti-mouse T- and B-Cell Activation Antigen (553666; dilution 1:500), BV786 anti-mouse IgD (563618; dilution 1:500), R718 anti-mouse CD45 (567075; dilution 1:500), BV711 anti-mouse Ki-67 (563755; dilution 1:500), BV786 anti-mouse CD95 (740906; dilution 1:500), BUV395 anti-mouse CD38 (740245; dilution 1:500), RB780 anti-Active Caspase-3 (570335; dilution 1:100), RB780 anti-mouse CD4 (568694; dilution 1:500), BUV563 anti-mouse CD3 (749277; dilution 1:500), BV421 anti-mouse CD185(562889; dilution 1:200), Biotin anti-mouse CD185 (551960; dilution 1:100), PE-Cy7 anti-mouse CD25 (552880; dilution 1:500), BUV805 anti-mouse I-A/I-E (748844; dilution 1:500), BV421 anti-mouse IL-10 (563276; dilution 1:100), BUV563 anti-mouse CD1d (741287; dilution 1:100), BV605 anti-mouse CD5 (563194; dilution 1:100), BV650 anti-mouse CD138 (564068; dilution 1:100), BUV737 anti-mouse Blimp-1(567834; dilution 1:100), BUV563 anti-mouse CD23 (741228; dilution 1:500), BUV737 anti-mouse CD21/CD35 (612810; dilution 1:500), BUV661 anti-mouse CD11b (612977; dilution 1:400). From BioLegend: PE-Cy7 anti-mouse/human GL7 Antigen (144620; dilution 1:500), Alexa Fluor 647 anti-mouse/human Bcl-6 Antibody (648305; dilution 1:200), BV605 anti-mouse CD86 (105037; Dilution 1:500), Streptavidin (B313199; dilution 1:200), PE anti-human/mouse/rat PCNA (2307908; 1:250), APC-Cy7 anti-mouse CD11c (117324; dilution 1:250); PE-Cy5 anti-mouse/human T-bet (644841, dilution 1:100). From ThermoFisher Scientific: APC anti-mouse CD4 (17-0041-81 dilution 1:500), PerCP-Cy5.5 anti-mouse FOXP3 (45-5773-82; dilution 1:250), eF450 anti-mouse IRF4 (48-9858-82; dilution 1:500), FITC anti-mouse CD279 (11-9985-85; dilution 1:200). From Biosearch Technologies: NP-PE (N-5070–1; dilution 1:200). Ghost Dye Violet 510 fixable viability dye (13-0870-T500; Tonbo Biosciences; San Diego, CA, USA) or DAPI (D3571; ThermoFisher Scientific) were used for the exclusion of dead cells. For intracellular markers, cells were fixed and permeabilized with the Foxp3/Transcription Factor Staining Buffer Set (501128857; ThermoFisher Scientific). Absolute cell numbers were calculated using CountBright Plus Absolute Counting Beads (C36995; ThermoFisher Scientific), and normalized to the weight of the corresponding tissue. When B-cell populations were sorted, single-cell suspensions of splenocytes were pre-enriched using an EasySep Mouse B Cell Isolation Kit (19854C; StemCell Technologies). The stated populations were then isolated using a FACSAria Fusion Flow Cytometer or FACSAria III Cell Sorter (BD Biosciences). Flow cytometry data was acquired on a FACSymphony A5 or FACSymphony SE cell analyzer, and analyzed using the FlowJo v10 software package (all from BD Biosciences).

#### Multiome data generation

Briefly, frozen cell suspensions were rapidly thawed in a 37°C water bath, washed with RPMI medium, centrifuged and counted. To enrich for B cells, cells were stained with a BV480 anti-human CD3 (566105; BD Biosciences) or a PerCP-Cy5.5 anti-human CD3 (45-0036-42; ThermoFisher Scientific) antibody, and DAPI. Live CD3-negative cells were subsequently sorted using a BD FACSAria III instrument, and nuclei from 250,000 sorted cells per specimen were isolated using the Low Cell Input Nuclei Isolation protocol for Single Cell Multiome ATAC + Gene Expression sequencing (CG000365 Rev C; 10× Genomics, Pleasanton, CA, USA). Transposition, GEM generation and barcoding, library pre-amplification, and ATAC and gene expression library construction were performed according to the Chromium Next GEM Single Cell Multiome ATAC + Gene Expression User Guide (CG000338 Rev G; 10× Genomics), using the Chromium Next GEM Single Cell Multiome ATAC + Gene Expression Reagent Bundle (1000283; 10× Genomics), in combination with the Chromium Next GEM Chip J Single Cell Kit (1000234; 10× Genomics), targeting a recovery of 5,000 nuclei. Gene expression and ATAC libraries were indexed using the Dual Index Kit TT Set A (1000215; 10× Genomics) and the Single Index Kit N Set A (1000212; 10× Genomics), respectively. Library quality was assessed using the High Sensitivity D1000 ScreenTape System (Agilent Technologies, Santa Clara, CA, USA). Libraries were sequenced using paired-end 75 nt reads on an Illumina NextSeq 550 platform (Illumina, San Diego, CA, USA).

#### Graphical abstract

Cartoons used in the graphical abstract were adapted from images in the SERVIER MEDICAL ART repository (https://smart.servier.com/). Use of these images falls within the terms of the Creative Commons Attribution 3.0 Unported License (https://creativecommons.org/licenses/by/3.0/).

### Quantification and statistical analysis

#### Multiome data analysis

The raw snMultiome data were pre-processed using Cell Ranger ARC (v2.02) (10× Genomics), which included cellular barcode demultiplexing, alignment to the GRCh38 reference genome, and quantification of RNA gene-barcode and ATAC fragment-barcode counts. CellBender (v0.3.0)^65^ was applied to identify cell-containing droplets and correct for ambient RNA contamination in RNA counts. Predicted doublets flagged by scDblFinder (v1.16.0)^66^ were excluded. We used Scanpy (v1.10.4)^67^ and SnapATAC2 (v2.8.0)^68^ for all downstream snMultiome data processing. Low quality cells were excluded if they exhibited low feature counts (<500 detected genes or <1,000 ATAC fragments), high mitochondrial gene content (>25%), or low transcription start site (TSS) enrichment scores (<5). The RNA gene–barcode matrix was normalized by dividing by total counts, scaling to 10,000 counts per cell, and applying a natural logarithm transformation with a pseudocount of one. An ATAC tile–barcode matrix was created by summing insertion counts within genome-wide 500-bp bins. The top 2,000 highly variable genes selected using scanpy.pp.highly_variable_genes with the seurat_v3 flavor, and the top 50,000 highly accessible tiles selected using snapatac2.tl.select_features were used for multi-modal dimensionality reduction. Integration of the RNA and ATAC profiles was performed using snapatac2.tl.multi_spectral. Subsequently, snapatac2.pp.harmony was used for batch-correction of the integrated embeddings, with donor as the batch covariate. The corrected embeddings were used to construct a neighborhood graph, followed by Leiden clustering and visualization with UMAP. Differentially expressed marker genes were identified using rank_genes_groups with Wilcoxon tests, and clusters were assigned to cell type identities based on canonical marker gene expression. For each cell type, sample-pseudobulk profiles were generated, to enable peak calling using snapatac2.tl.macs3. A set of non-overlapping 500-bp peaks was created using snapatac2.tl.merge_peaks. Chromatin accessibility deviation scores were calculated for single cells using chromVAR (v1.24.0).^16^ NFKB2 and RELB B cell ChIPseq datasets were curated from the ChIP-Atlas database. The NC NF-κB signature scores were calculated using scanpy.tl.score_genes.

#### Statistical analysis

Statistical parameters, including value and definition of n, precision measures, tests used, and significance are reported in figures and/or figure legends. Cohort sizes were derived from power calculations based on pilot assays or reports using similar approaches/models,^8^^,^^17^^,^^43^^,^^63^ whenever available. Statistical analysis was conducted using Prism 10 (GraphPad Software, San Diego, CA, USA), or R/Pthyton language scripts and specified packages. In particular, the multiome analysis was performed using R (v4.3.3) and Python (v3.11). Normality was tested before applying parametric tests. If assumptions were violated, appropriate transformations (e.g., log transformation) or non-parametric tests were applied, to ensure valid statistical inference. Data was deemed to be statistically significant if *p* < 0.05. Asterisks in figures represent statistical significance (∗, *p* < 0.05; ∗∗, *p* < 0.01; ∗∗∗, *p* < 0.001).

## Supplemental information

Document S1. Figures S1–S5

Table S1. Differential ChIP-Atlas TFBS accessibility score testing
