# Supplementary material for: Non-canonical NF-κB drives a fate switch from germinal center to early effector B cells
Source: iScience. 2026 Jul 22;29(8):116917. doi: 10.1016/j.isci.2026.116917 (PMC13426217; doi:10.1016/j.isci.2026.116917)
Supplement: Document S1. Figures S1–S5 [file mmc1.pdf]

iScience, Volume *29*

Supplemental information

Non-canonical NF-κB drives a fate switch from germinal center to early effector B cells

Waqas Nawaz, Amos Fong, Miguel P. Cardoso, Jing Tang, Jenny Zhao, Michael Y. Li, Shinya Rai, Aidan Beresford, Waleed Alduaij, Merrill Boyle, Christian Steidl, David W. Scott, and Leandro Venturutti

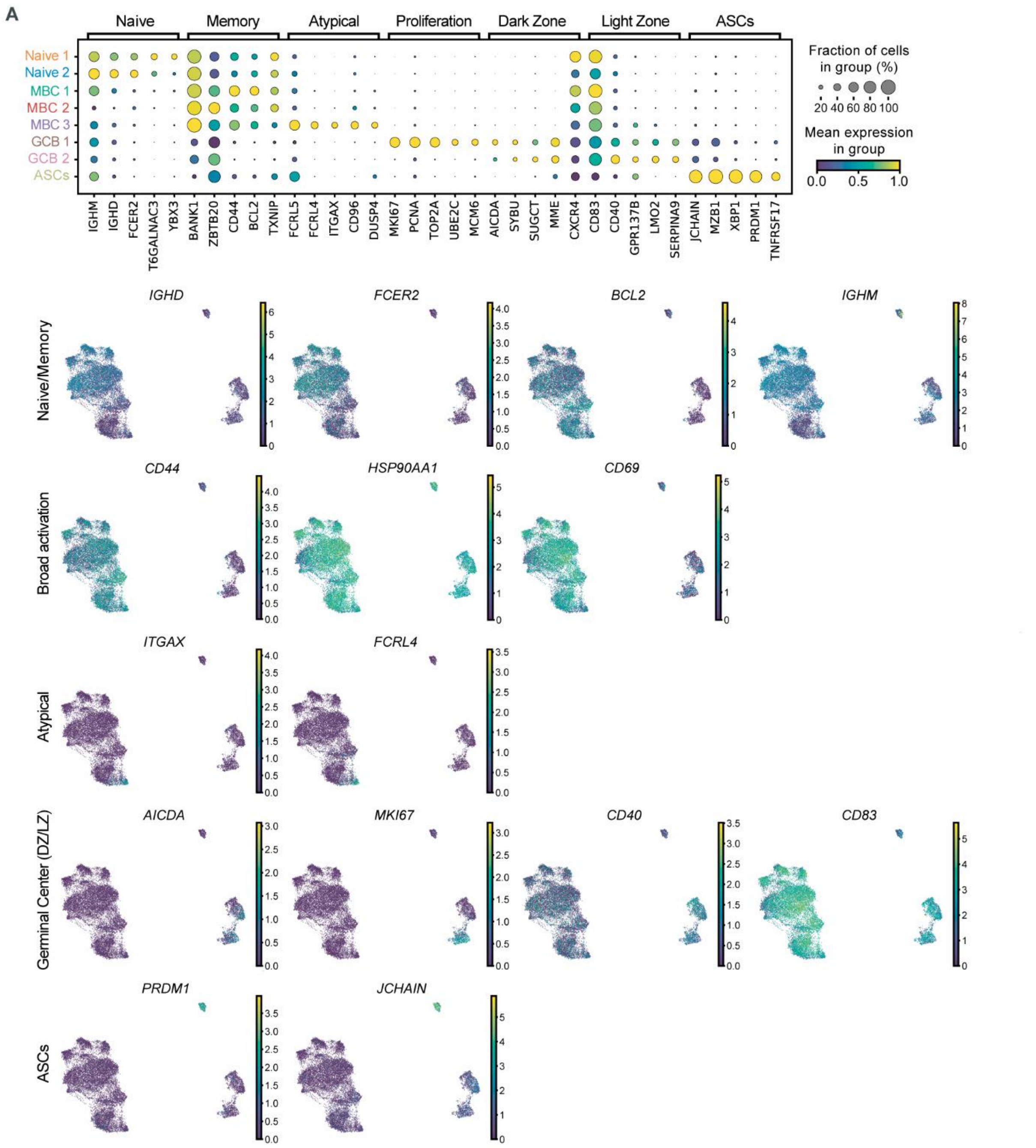

SUPPLEMENTAL FIGURE 1. Marker gene expression across tonsillar B cell subsets. ***Related to Figure 1.***

(**A**) Top: Dot plot showing relative expression levels for selected genes of interest in B cell subsets from **Figure 1B**. Bottom: UMAP plots illustrating relative expression for selected genes of interest in B cell subsets from **Figure 1B**.

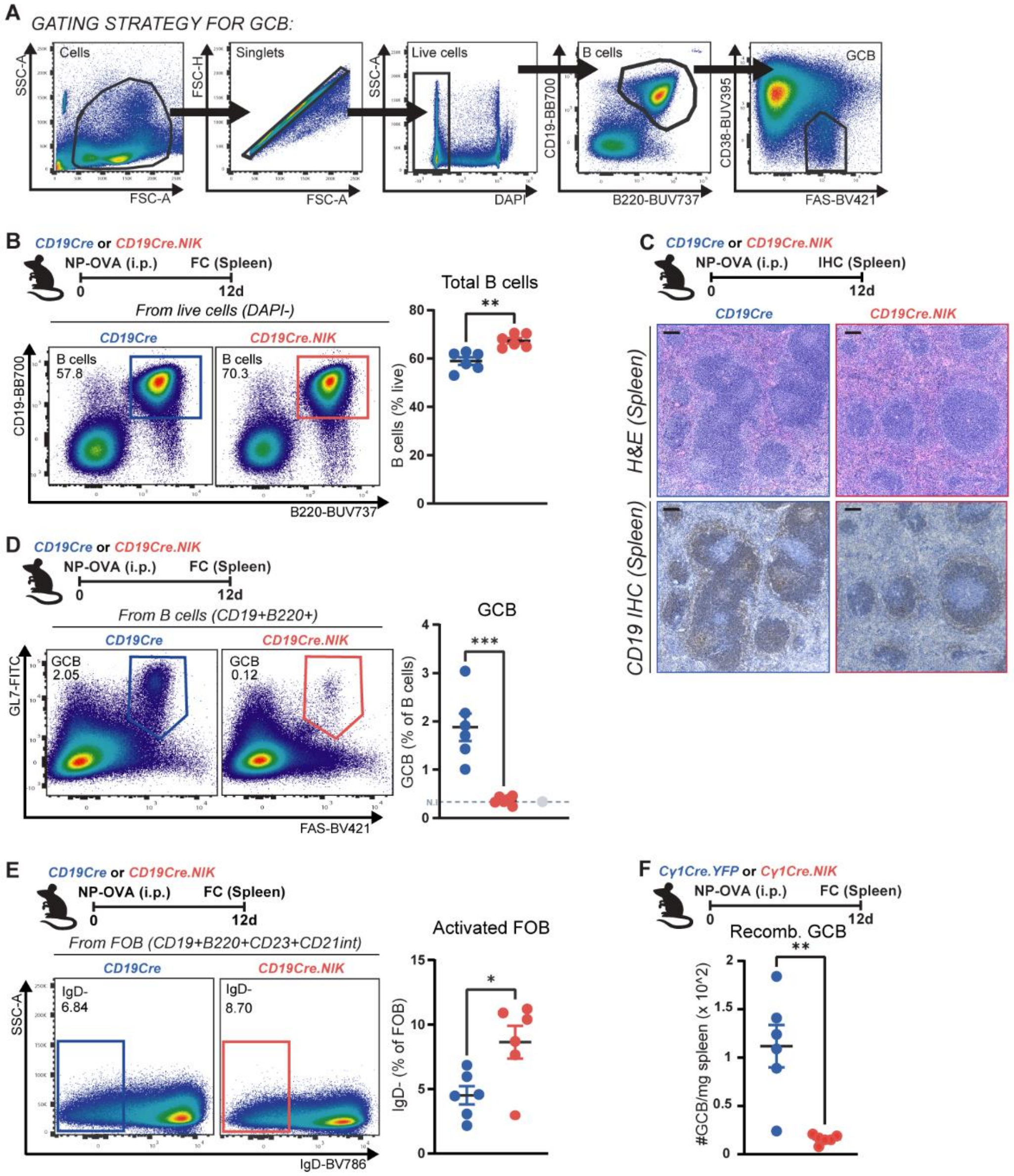

SUPPLEMENTAL FIGURE 2. Profiling of altered splenic B cell subsets. ***Related to Figure 2.***

(**A**) Representative full FC gating strategy for **Figure 2A**.
(**B**) FC analysis of total B cells for experiment in **Figure 2A**. *CD19Cre.NIK*: *CD19Cre;R26NIK*^{GFP}.
(**C**) H&E and CD19 IHC on splenic sections from animals in **Figure 2C**. Scale = 100µm.
(**D**) FC profiling of GCB using an alternative gating strategy to that in **Figure 2A**. N.I: non-immunized control animal.
(**E**) FC analysis of IgD expression in splenic B cells from animals treated as in **Figure 2A**.
(**F**) FC-derived GCB cell counts from animals treated as in **Figure 2D**. *Cγ1Cre.YFP: Cγ1Cre;R26YFP; Cγ1Cre.NIK: Cγ1Cre;R26NIK*^{GFP}.

Individual dots represent biological replicates. Values represent mean ± SEM. Data reproducible with two repeats. Here and elsewhere, value distributions were assessed using the Shapiro–Wilk normality test, and parametric or non-parametric statistical tests were applied accordingly. *p<0.05; **p<0.01; ***p<0.001, using unpaired two-tailed Student's t-test (**B, D-F**).

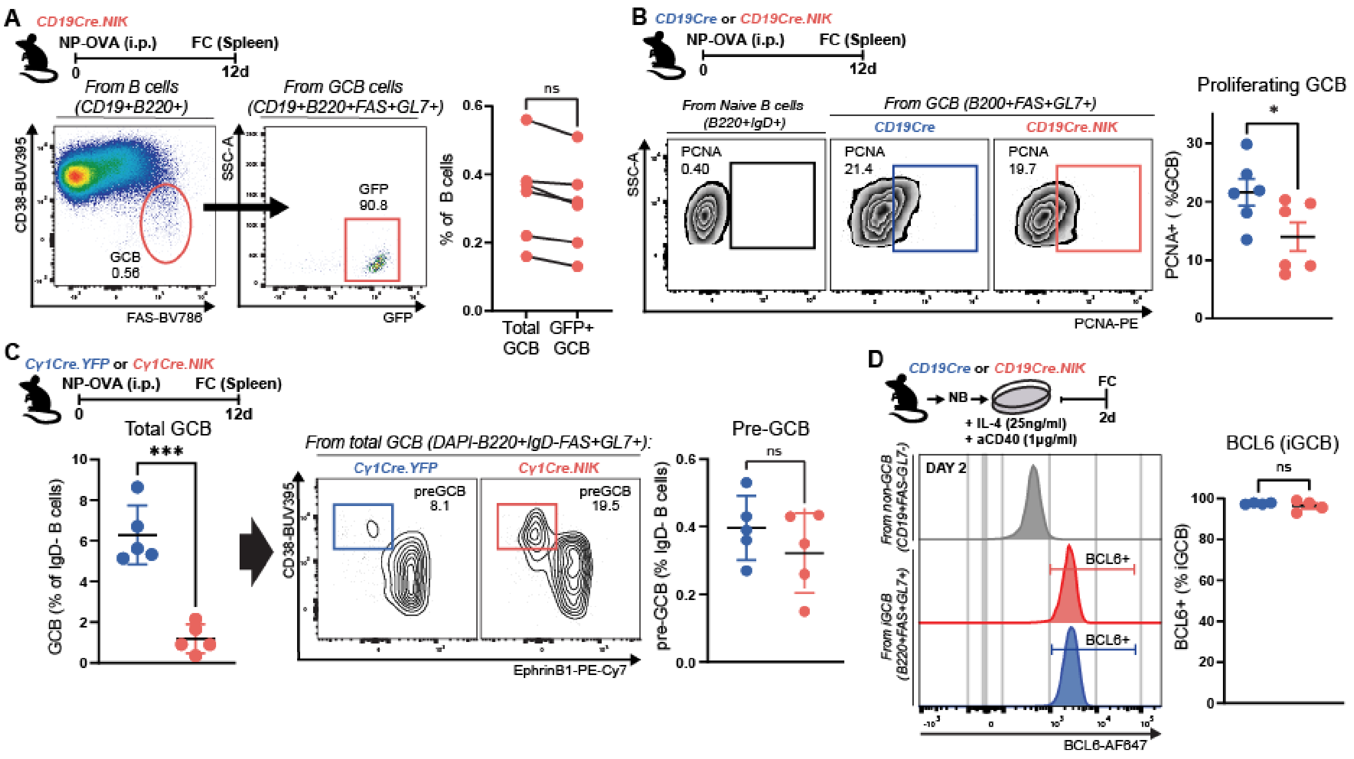

SUPPLEMENTAL FIGURE 3. GCB dynamics and intracellular markers. ***Related to Figure 3.***

(**A**) FC-based comparison of total and recombined (GFP+) GCB relative abundances.
(**B**) FC analysis of proliferating GCB abundance, based on intracellular PCNA expression.
(**C**) FC analysis of (left) total splenic GCB pool and (right) precursor GCB population.
(**D**) FC profiling of intracellular BCL6 expression in iGCB from **Figure 3D**.

Individual dots represent biological replicates. Values represent mean ± SEM. Data reproducible with two repeats. NS, not significant; *p<0.05; ***p<0.001; using paired (**A**) or unpaired two-tailed Student's t test (**B**), or Mann–Whitney U-test (**C-D**).

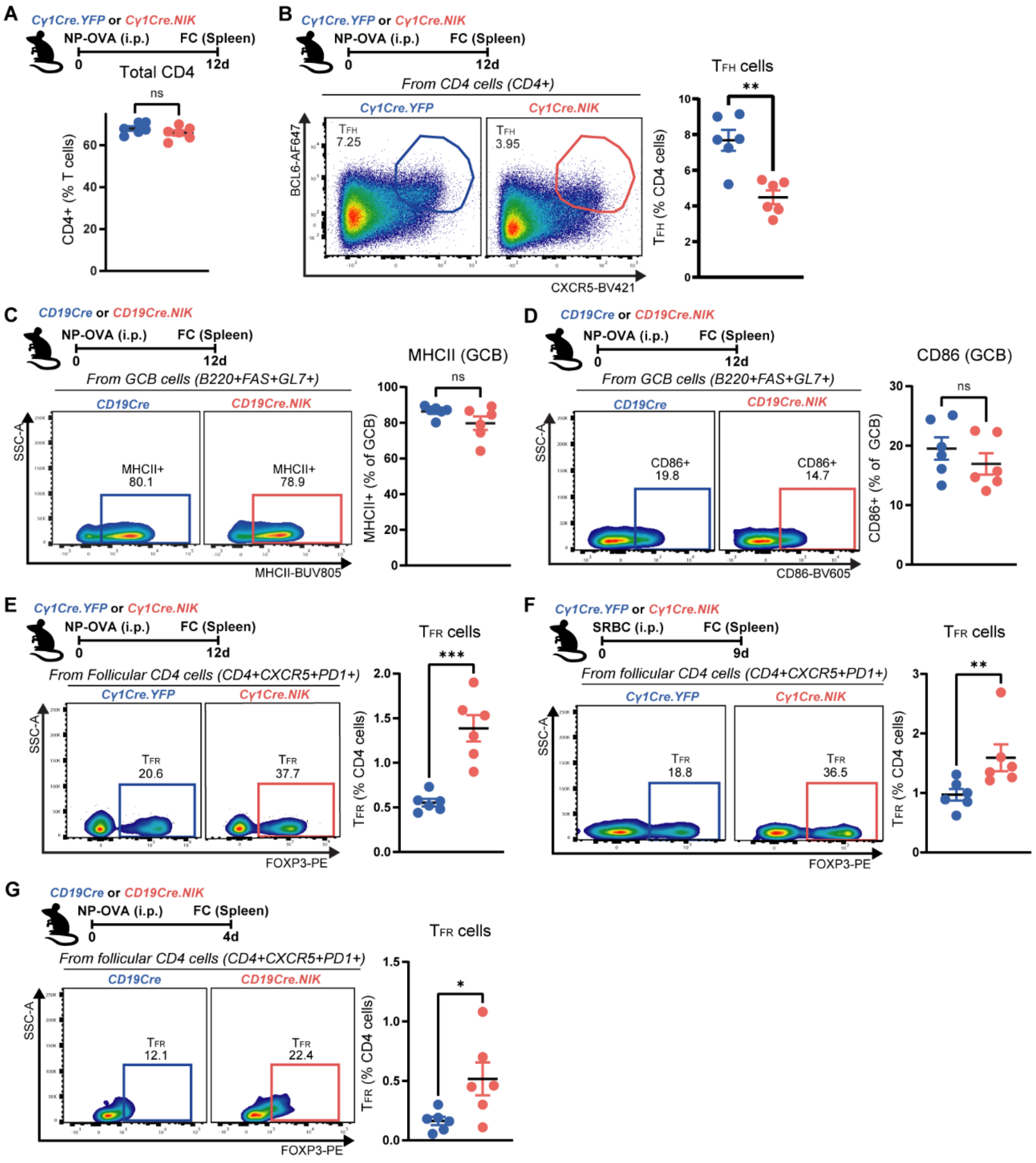

SUPPLEMENTAL FIGURE 4. T_{FR} dynamics and GCB activation. *Related to Figure 4.*

(**A**-**B**) FC analysis of (**A**) total CD4 cells, or (**B**) T_{FR} cells using an alternative CRE model to that in **Figure 4C-D**.
(**C** and **D**) FC profiling of (**C**) MHCII or (**D**) CD86 expression in GCB from animals treated as in **Figure 2D**.
(**E** and **F**) FC analysis of T_{FR} frequency using (**E**) an alternative CRE strain and/or (**E**) an alternative TD antigen to those in **Figure 4F**.
(**G**) FC analysis of T_{FR} at an earlier timepoint than in **Figure 4F**.

Individual dots represent biological replicates. Values represent mean ± SEM. Data reproducible with two repeats. NS, not significant; *p<0.05; **p<0.01; ***p<0.001, using unpaired two-tailed Student's t-test (**A-E**), or Mann–Whitney U-test (**F, G**).

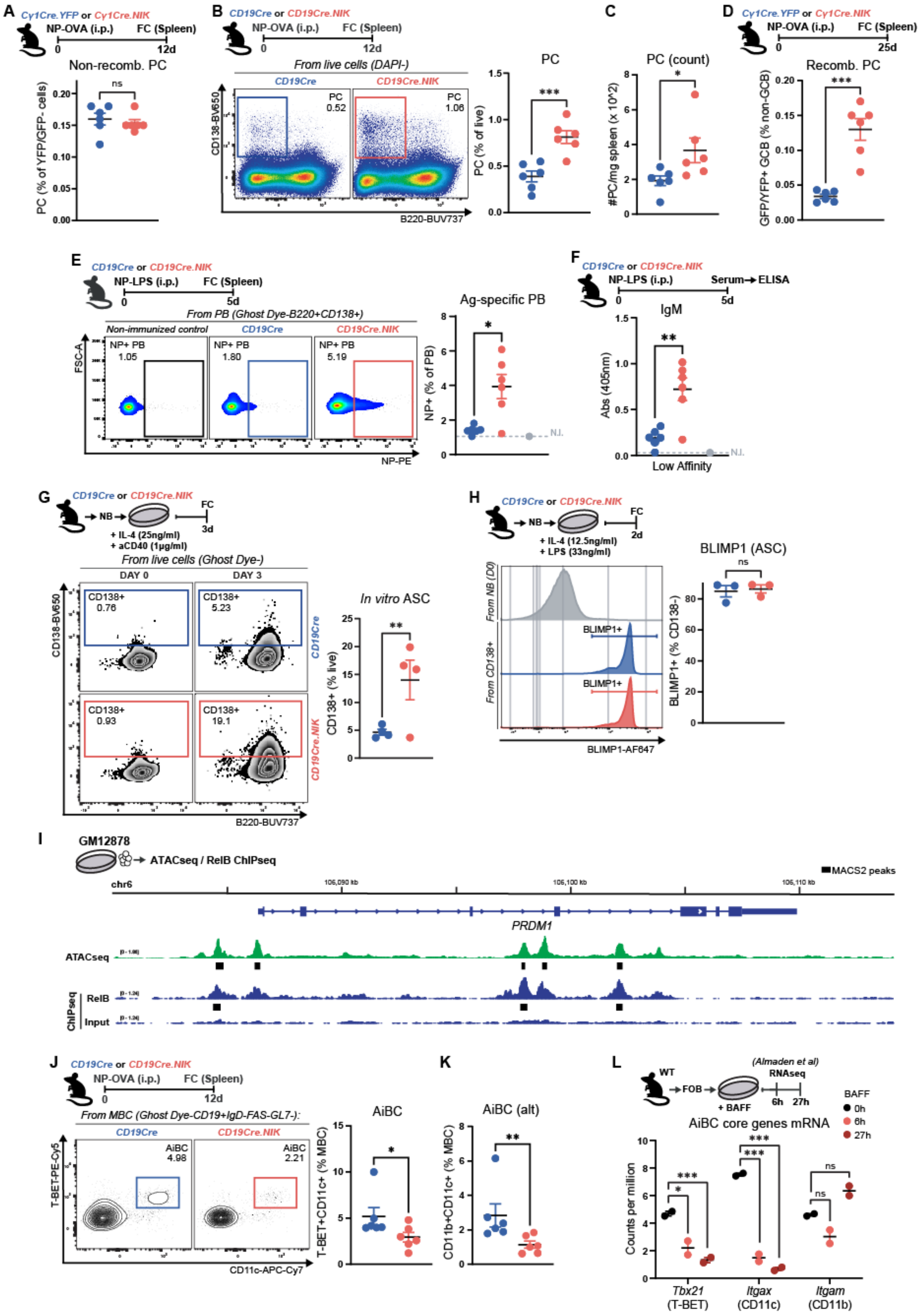

A
Cγ1Cre.YFP or Cγ1Cre.NIK
NP-OVA (i.p.)
FC (Spleen)
Non-recomb. PC
PC (% of YFP/GFP- cells)
B
CD19Cre or CD19Cre.NIK
From live cells (DAPI-)
CD138-BV650
B220-BUV737
PC
C
PC (count)
#PC/mg spleen (x 10^2)
D
Recomb. PC
GFP/YFP+ GCB (% non-GCB)
E
NP-LPS (i.p.)
From PB (Ghost Dye-B220+CD138+)
Non-immunized control
NP+ PB
FSC-A
NP-PE
Ag-specific PB
NP+ (% of PB)
F
Serum→ ELISA
IgM
Abs (405nm)
Low Affinity
G
+ IL-4 (25ng/ml)
+ aCD40 (1μg/ml)
From live cells (Ghost Dye-)
DAY 0
DAY 3
CD138+
In vitro ASC
CD138+ (% live)
H
+ IL-4 (12.5ng/ml)
+ LPS (33ng/ml)
BLIMP1 (ASC)
BLIMP1+
BLIMP1-AF647
BLIMP1+ (% CD138-)
I
GM12878
ATACseq / RelB ChIPseq
MACS2 peaks
chr6
PRDM1
ATACseq
ChIPseq
RelB
Input
J
From MBC (Ghost Dye-CD19+IgD-FAS-GL7-):
T-BET-PE-Cy5
CD11c-APC-Cy7
AiBC
T-BET+CD11c+ (% MBC)
K
AiBC (alt)
CD11b+CD11c+ (% MBC)
L
WT
(Almaden et al)
RNAseq
+ BAFF
BAFF
AiBC core genes mRNA
Counts per million
Tbx21
(T-BET)
Itgax
(CD11c)
Itgam
(CD11b)

SUPPLEMENTAL FIGURE 5. ASC and atypical B cell phenotypes. *Related to Figure 6.*

(**A**) FC analysis of non-recombined (GFP/YFP-) antibody-secreting cells in animals from **Figure 6A**.
(**B** and **C**) FC analysis of total ASC, presented as (**B**) relative frequency or (**C**) absolute cell numbers, using an alternative CRE system than in **Figure 6C**.
(**D**) FC analysis of recombined (GFP/YFP+) PC at a later timepoint than in **Figure 6C**.
(**E**) FC analysis of antigen-specific (NP+) PB, using T-independent antigen.
(**F**) ELISA results for NP-specific serum antibodies, from animals in (**E**). Abs: absorbance.
(**G**) FC profiling of *in vitro* derived ASC, from cells treated as in **Figure 3E**.
(**H**) FC analysis of intracellular BLIMP1 expression in CD138+ cells from the experiment in **Figure 6G**. Staining results in naive B cells (NB) are shown as a negative control.
(**I**) ChIPseq analysis of RelB binding at the *IL10* gene locus [1]. An ATACseq track is included to indicate accessible regions in the same cell line. Representative tracks are shown. Black boxes indicate significant peaks called by MACS2 [2].
(**J**) FC analysis of splenic AiBC population.
(**K**) Same as in (**J**), but using an alternative combination of markers.
(**L**) RNAseq analysis of AiBC core markers expression levels in the indicated model (GSE62559).
Individual dots represent biological replicates. Values represent mean ± SEM. Data reproducible with two repeats. NS, not significant; *p<0.05; **p<0.01; ***p<0.001, using unpaired two-tailed Student's t test (**A-D**), or Mann–Whitney U-test (**E-H**, **J-K**), or a Benjamini–Hochberg-corrected Wald test (**L**).

SUPPLEMENTAL REFERENCES

1. Zhao, B., Barrera, L.A., Ersing, I., Willox, B., Schmidt, S.C., Greenfeld, H., Zhou, H., Mollo, S.B., Shi, T.T., Takasaki, K., et al. (2014). The NF-κB genomic landscape in lymphoblastoid B cells. Cell Rep *8*, 1595-1606. 10.1016/j.celrep.2014.07.037.
2. Feng, J., Liu, T., Qin, B., Zhang, Y., and Liu, X.S. (2012). Identifying ChIP-seq enrichment using MACS. Nat Protoc *7*, 1728-1740. 10.1038/nprot.2012.101.
